# Multi-level meta-analysis of whether fostering creativity during physical activity interventions increases their impact on cognitive and academic outcomes during childhood

**DOI:** 10.1038/s41598-023-35082-y

**Published:** 2023-05-24

**Authors:** Fotini Vasilopoulos, Holly Jeffrey, Yanwen Wu, Iroise Dumontheil

**Affiliations:** 1 Centre for Brain and Cognitive Development, Department of Psychological Sciences, Birkbeck, University of London, Malet Street, London, WC1E 7HX UK; 2 Centre for Educational Neuroscience, University of London, London, UK; 3 University of Cambridge, Cambridge, UK

**Keywords:** Psychology, Human behaviour

## Abstract

Neuroplasticity research supports the idea that varied practice and new environments promote cognitive engagement and enhance learning. Expanding on a meta-analysis of the effect of physical activity interventions on cognition and academic outcomes, we reviewed and quantified the impact of task and environmental factors that foster creative physical activity. Interventions were considered as fostering creative physical activity to a greater extent if (1) they were varied, (2) relied less on technical acquisition, instruction or demonstration, (3) involved open spaces, props, or open-ended instructions, and (4) involved interactions with peers. A wide range of physical activities were considered, from dance to aerobic exercise across 92 studies in 5–12-year-old children. Creativity ratings of physical activity interventions were varied but did not associate with greater beneficial effects on executive functions (*k* = 45), academic achievement (*k* = 47), or fluid intelligence (*k* = 8). Studies assessing on-task behaviour (*k* = 5) tended not to foster creativity, while reversely studies assessing creativity tended to foster creative physical activities (*k* = 5). As a group, three studies that fostered more creative PA showed a small significant negative summary effect on cognitive flexibility. Considering qualitative differences in the physical activities performed in schools will improve our understanding of their mechanisms of impact. Future research should consider using more varied measures, including more proximal outcomes that involve body movements (e.g., a Simon Says task to measure inhibitory control).

## Introduction

Recently researchers have recommended a focus on the qualitative aspects of physical activity (PA) that could support cognitive development rather than on the quantity characteristics, such as intensity and duration of PA sessions^1,2^. Physical activity researchers have applied the concept of “cognitively engaging” PA to highlight a path through which PA may have an impact on cognition over and beyond potential direct benefits of regular exercise on the neural substrate underlying cognition^3–5^. The suggestion here is that it is not only the quantity of exercise but also the type of cognitive effort engaged during PA that is key to enhancing cognitive skills^1^. One particular focus has been the impact of cognitively engaging PA on executive functions (EF), a set of cognitive processes that allow the regulation of attention and behaviour when automatic responses are inappropriate^6^.

Two theories propose cognitive explanations for beneficial effects of PA. First, the skills acquisition theory postulates that the motor and cognitive complexity of PA influence cognitive processes^7^. For example, PA can be considered cognitively engaging when it requires complex movement patterns rather than simple repetitive movements. It has been suggested that the response to practicing complex tasks may interact with the response to the level of physical effort required^7^. Second, the theory of embodied cognition emphasises the importance of grounding cognition in the body and suggests that mental processes are supported by interactions between the body, the brain and the external environment^8^. Two meta-analyses have investigated the hypothesis that cognitively engaging PA leads to greater cognitive benefits that other types of PA by comparing randomised controlled trials (RCT) of (a) aerobic, (b) motor skills and (c) cognitively engaging PA interventions^2,9^. Both studies found that physical activities with greater cognitive engagement, for example those requiring greater attention, remembering rules and constantly thinking of action plans, involving academic content or frequent rule changes, emphasising variability and/or integrating social and emotional skills, have greater positive effect on executive functions than those with lower cognitive engagement.

Some physical activities include components that engage specific cognitive skills in addition to the physical exercises^2,10^. For example, activities like dance incorporate a creativity element. It has been suggested that creativity is a key factor for doing well in life^11^ and that it is essential for our future society^12,13^. Creativity can help students solve problems and challenges outside an educational context. Creativity will be vital for jobs in the future as a result of rapid technological advancement^14^. Today’s children will most likely be employed in roles that do not currently exist, applying new technology such as artificial intelligence. Creating an education environment that harnesses children’s capacity to innovate will help in their journey to navigate this uncertainty. Some have shown that younger children perform better than college students on a creative problem-solving task^15^. While creativity has been studied in children, in particular with regards to play, there is currently no clear-cut pattern of changes in creativity over the school of childhood and adolescence^16–22^. Some suggest that these mixed results are influenced by life experiences and/or environmental experiences^19^.

An embodied approach to creativity focuses on movements and interactions with the environment^23^ and a promotion of exploration and originality^24^. Creative practice as a mean to train cognition has also been studied through non-physical interventions for primary school-aged children. Specifically, different art interventions including music^25,26^, drama^27^ and visual art^28^ have shown evidence of promise. We therefore propose that creativity added to a PA intervention could increase positive impacts on cognitive and metacognitive processes through additive or moderating effects. Interest in the effects of physically creative practices on outcomes is recent; a few studies applying creative dance interventions on children have indicated positive influence between dancing and a range of cognitive measures^29–31^.

### Creative movement

Creative movement can be described as a “functional and original movement solution to achieve a task goal”^32^. As architects of the learning environment, teachers are in a position to promote exploration of movement^24^ and can offer children meaningful problem-based activities in authentic movement contexts^33,34^. Pathways to creativity in movement for children are supported by the opportunities offered in their environment^35,36^. The motor learning environment is shaped by what is taught, where it is taught and how it is taught. Tools such as improvisation and active open-ended problem-solving instructions in relation to movement, using a non-judgmental approach, open the window to experimentation and thus creativity^37^. Creative movement in an educational context puts the child “in charge of the task they are performing”^32^, which could promote cognitive engagement, as well as self-regulation, an important element supporting embodied cognition^38^.

Neuroplasticity research supports the idea that varied practice and new environments promote cognitive engagement^3^ and enhance learning^39,40^. Moreau and Conway^41^ identified three aspects of physical (motor) activities which may increase neuroplasticity and therefore learning: novelty, diversity and task complexity. A teacher has several tools to facilitate a dynamic environment through varied practice, real-world activities and diverse situations, all of which can promote a creative environment and ultimately support cognitive processes^10,38,42^.

### Physical activity and pedagogical frameworks

Traditionally, PE has been taught in an operational and rigid way, with limited theoretical or pedagogical considerations^43,44^. Two pedagogical models have been proposed recently in the literature to frame teaching PE in the curriculum^36^. Both types of pedagogy can be used in physically active lessons, and both play an important role at different stages of motor skill learning. First, linear pedagogy involves the acquisition of technical skills, which is achieved through constant or block training using drills or fixed choreography that are reproduced until the motor skill is mastered^36^. To master the skill, the movement is broken down into steps and any variability to the pattern is limited so that the participant can acquire the optimal way of moving^36,45^. The learner progresses through acquiring movement knowledge in a linear process—that is the movement pattern will be erratic and have errors and is repeated until variability is reduced and the movements are finally performed in an automatic way^46^. Linear pedagogy may align with the natural scaffolding of learning by executive function in the early phases, until automaticity is achieved^36^.

Non-linear pedagogy involves the teacher adjusting the task, the environment, or both, to engage the participant^36,45^. Task adjustment may be done by varying the task itself, or by adjusting the environment in which the task is performed. The task may be varied by changing the rules of a game or altering the number of participants in the task. Instructions can also be used to adjust the task by using open-ended problem-solving instructions, such as ‘Show me any kind of…’, ‘Show me any other way to…’, or ‘How would you do that differently?’^36^. The environment can be adjusted by changing the size of the space or changing the equipment. For example, you could move from an outdoor space such as a school field to an indoor space with or without boundaries, or introduce different props, such as different music in dance or a range of different balls in sports. Modifying the task and/or environment may support the development of executive functions through inhibiting routine movement patterns (inhibitory control) or flexibly switching between variable tasks (cognitive flexibility).

Cognitive development is sensitive to social and environmental influences^47,48^. Some have suggested that linear teaching practices are overly structured and do not consider individual differences^49^. In this way teaching strategies in linear pedagogy is mainly teacher-centred^50^. Non-linear teaching practices can be more student-centred if applied appropriately; they can make PA more complex in nature and more cognitively engaging. Both skills acquisition and embodied cognition theories imply that our motor behaviours are part of a self-organisation process at play when interacting in social situations and responding to environmental features^51^. Non-linear pedagogy reflects this understanding by incorporating teaching strategies that develop a relationship between the participant and the environment^33,52,53^. Non-linear pedagogical teaching practices can be used in the younger years especially if they are based on children’s prior knowledge of basic movement patterns such as walking, running, jumping^54,55^. Engaging children in solving open-ended movement problems will give them space to express creativity and practice self-regulation skills for emotions and behaviour^36^.

Building on a meta-analysis assessing the effects of a range of PA intervention on executive functioning, fluid intelligence, on-task behaviour, creativity and academic outcomes in primary school children^56^, the aim of the present analyses is to extent the work to examine whether PA interventions that fostered creativity to a greater extent led to greater cognitive and academic improvements. Interventions were classified based on a series of task- and environment-based characteristics that have been identified in existing cognitive and pedagogical motor learning literature as supportive of a creative physical practice (see Table 1 and Online resource Table S1 for more detail). Interventions which involved more varied and divergent tasks, and activities carried it out in groups, as a team or against an opponent, were considered to foster creativity more than interventions which involved more repetitive, convergent tasks carried out individually and without an opponent. In addition, interventions where the teacher encouraged structured improvisation and problem-solving, in a flexible space, were considered to foster creativity more than interventions where the teacher focused on technical instructions and demonstrations in a fixed environment^57^.

**Table 1:** Creativity characteristics used to classify the physical activity interventions.

	Lower creativity	Higher creativity	Literature background
Task characteristics	Repetitive	Varied	Training where children are presented with more varied situations influences cognition more than blocked or repetitive training^58^
	1. Individual 2. Parallel 3. No opponent	1. Group 2. Team 3. Opponent	Working with someone can bring diversity to the task and lead to the generation of different movements^58–60^
	Technical acquisition	Real world activity	Prescriptive tasks such as drills are set and reproduced until the motor skill mastered. This leaves little room for cognitively stimulating generation of novel movement solutions^61^
	Convergent task	Divergent task	A divergent task allows for exploration as the child generates different movement sequences^57,62^
Environment	Technical instruction and demonstration	Open-ended instruction	Demonstrating and instructing choreography in dance or a particular skill in sport activities (e.g. tennis racket swing) limits the number, variability and uniqueness of movement solutions compared to structured improvisation or problem-solving instructions^57,58^
	Fixed space	Open space	A fixed space (court, swim lanes, track) limits the possibility of movement variability^63^. A flexible space, particularly outdoor space improves inhibitory control^10^. An indoor space with changing boundaries or no boundaries also offers other movement possibilities^64^
	No props	Props	Using props (e.g., music in dance or balls in sports) places demands on cognitive processes as the participant needs to respond to cues that are actively changing^38^

Recently, some physical activity interventions have been designed to incorporate elements that would foster creativity in children, moving away from repetitive aerobic physical activities, either because researchers were interested in improving children’s creativity^30,57^, or to make the interventions more cognitively engaging^32,36^. However, there has not been systematic research into whether fostering creativity in movement is a way to boost cognitive engagement and improve children’s cognitive and educational outcomes. This meta-analysis therefore complements other meta-analytic reviews^2,9,65^ by specifically quantifying the potential beneficial impact of fostering creativity during physical activity interventions.

## Methods

### Study selection

This systematic review and meta-analysis was performed according to PRISMA guidelines^66^ and methods were pre-specified and documented in advance in a protocol that was published on the Open Science Framework database for preregistered reviews (https://osf.io/uvpb4). The primary search included seven electronic databases: PubMed, Education Resources Information Center, British Education Index, Australian Education Index, Applied Social Sciences Index and Abstracts, Web of Science, and PsycINFO. Key terms related to executive functioning, academic achievement, physical activity interventions, and children (see Online resource Table S2). The focus was on peer reviewed randomised control trials in typically developing 5–12-year-old children using objective outcome measures published between 01/01/2000 and 30/09/2022 (see Online resource Table S3 for more detail about selection criteria and our companion paper^56^.

### Data extraction

The creativity of the included studies was assessed by two reviewers (FV and YW). Study characteristics were grouped in two main categories: task and environment^36,52^, themselves split into sub-categories described in Table 1. Each category was rated between 0 and 2; more detail about these ratings is provided in the Online resource Table S1. When insufficient information was available authors were contacted in the first instance. When sufficient information was still not available no rating was entered for a given sub-category. Sub-categories had missing information for 0–4% of studies, except for the type of instruction (technical and demonstration vs. open-ended) where 16% of studies did not provide this information. To allow for this missing information, available ratings on each sub-category were then averaged to provide a combined “creativity” score. This average creativity score was then scaled by seven to reflect the full-scale score, resulting in a range between 0 (no creative element) to 14 (maximum number of creativity elements). The inter-rater reliability score (Cohen's Kappa) was 0.52, indicating moderate agreement^67^.

Intervention duration categories were classified as ≤ 6 weeks, 7–10 weeks, 11–24 weeks or ≥ 25 weeks. The frequency categories of PA sessions per week were categorised as 1, 2, 3 or > 3 sessions per week. The duration of training sessions were categorised as < 20 min, 20–44 min, 45–60 min or > 60 min. These categories were chosen based on the duration of the interventions included in the meta-analyses, which themselves reflect the type of intervention taught (e.g., PA breaks and PA with academic instruction tended to be brief, 10–20 min) and the typical duration of PE classes (45–60 min). Indeed, primary school PE provision tends to be around 100 min per week worldwide^68^, and PE classes tend to be split into two sessions (e.g. in England, Ofsted, 2022^69^). Classification of teacher qualification was based on a recent meta-analysis investigating the quantity and quality of PE interventions during childhood^70^. Classroom teachers and researchers were classified as having lower professional qualifications in relation to PE teaching skills, while exercise science researchers and PE teachers were considered as having higher professional qualifications. Intensity of PA was categorised based on ratings provided in the studies and used in sports literature: low, low to moderate, moderate, moderate to vigorous or vigorous^71,72^. When insufficient information was available authors were contacted in the first instance. When sufficient information was still not available a “cannot tell” rating was given. Finally, duration of effects was categorised as short term (< 2 weeks), medium term (2–12 weeks), and long-term (> 12 weeks). We considered immediate effects within a two-week period due to the difficulty of collecting data immediately post intervention in a school setting. Full details of data extraction are described in our companion paper^56^.

### Data analysis

Statistical analyses were performed in R (v.3.3.2) using the rma.mv function of the metafor package^73^. We used an alpha level of 0.05 and report 95% CI. First, we performed independent *t*-tests or ANOVAs to test whether the creativity ratings of the interventions associated with key other characteristics of the interventions. Second, a three-level multilevel meta-analytic approach was used to handle non-independent effect sizes and nested effect sizes, e.g., when including more than one measure from a single study^74^. This is a deviation from our planned analyses; we decided to adopt this novel approach which allows the inclusion of a larger number of individual effect sizes to strengthen research on the impact of PA interventions. We decided to include a single measure per task to avoid having another level, “task”, within studies.

**Figure 1:**
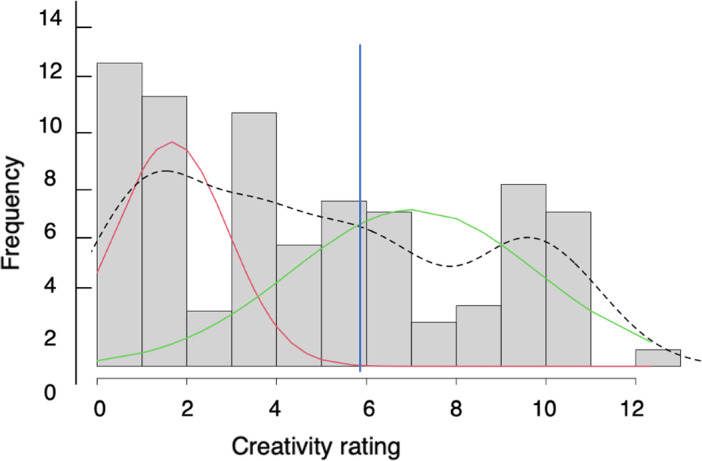
Bimodal distribution of interventions creativity scores in the studies included in the meta-analysis. Note: The red and green lines plot the distribution of creativity ratings for the lower and higher creativity sub-groups respectively. The black dotted line plots the overall distribution. The blue vertical lines indicate the cut-off identified through finite mixture model using the Expectation–Maximization algorithm.

Two sets of analyses tested whether the extent to which interventions fostered creativity influenced the impact of the interventions. First, meta-regressions investigated whether the effect size of PA interventions associated with their creativity ratings. The distribution of creativity ratings is shown in Fig. 1. Hartigans’ dip test for multimodality indicated that the distribution of creativity scores was not unimodal (*D* = 0.05, *p* < 0.01), with two apparent clusters. Finite mixture model using the Expectation–Maximization (E-M) algorithm confirmed the two distributions, with a cut-off of 5.93. Therefore, in a second step, interventions were grouped as lower or higher creativity PA interventions based on this cut-off. Moderator analysis compared whether higher creativity PA interventions, as a group, showed larger effect sizes than lower creativity PA interventions. Even in the absence of differences, exploratory subgroup analyses were run to estimate summary effect sizes for higher and lower creativity studies separately, which may reduce heterogeneity. In effect this allowed us to carry out meta-analyses focusing on higher creativity studies, which can serve as a reference point in future work. Analyses were first conducted on grouped EF outcomes and AA outcomes, and on fluid intelligence, creativity and on-task behaviour measures. On-task behaviour refers to verbal or motor behaviour that relates to the learning activity^75^. Then further analyses considered each EF and AA sub-domain separately: mathematics, language, attention, cognitive flexibility, inhibitory control, planning, and working memory.

There is currently no agreement on the number of effect sizes to be included in meta-regression analysis. Although the Cochrane handbook suggests a minimum of ten studies for each sub-category without providing support for their recommendation^76^, others suggest a minimum of six studies for a continuous variable, and four in each subgroup for a categorical variable^77^. Thus, meta-regressions were completed when there were at least six effect sizes and the higher/lower creativity group analyses when there were at least four effect sizes per group. If creativity showed a significant association, further meta-regressions were conducted controlling for characteristics of the interventions/studies that correlated with creativity ratings to investigate whether associations remained significant^78^. Effect sizes were classified as small (0 ≤ *g* ≤ 0.50), moderate (0.50 < *g* ≤ 0.80) or large (> 0.80)^76^. Sensitivity analysis was run excluding influential studies which were identified by calculating Cook’s distance and using *F*_0.5_(*p, n* *−* *p*) as a cut-off and by checking the Cook’s distance plots when creativity rating was included as a predictor^79^. Confidence intervals are reported to identify bias due to small samples and categorised as low (< 30%), moderate (50%) and high (> 75%) when reporting heterogeneity for *I* ^2^^81–83^. Additional information on statistical techniques are given in our companion study^56^.

### Study quality

Each study was rated as weak, moderate or strong on six components: selection bias, study design, confounders, blinding, data collection methods, withdrawals, and drop-outs. The ratings of the risk of bias assessment, using the EPHPP Quality Assessment Tool for Quantitative Studies^83^, are provided for each study in Online resource Table S4. Analyses and discussion of these results are shown in our companion study^56^.

## Results

The companion meta-analysis identified 92 studies. In brief, PA interventions as a whole were found to lead to improvements in working memory, fluid intelligence, on-task behaviour, and creativity. However, heterogeneity was high, with low to moderate certainty of evidence. Moderation analyses indicated that moderate to vigorous and/or aerobic PA interventions benefited EF, while PA with academic instruction benefited mathematics outcomes and enriched PA benefited language outcomes^56^. The focus of the present analyses was on whether qualitative aspects of the PA interventions that may foster creative movement may account for differences in effect size between studies. The “Results” section provides a description of study characteristics, followed by intervention characteristics and meta-analytic findings of the meta-regressions with intervention creativity rating as predictor of differences in effect sizes. The main study characteristics and effect direction of all outcome measures are presented in Online resource Table S4. Results of comparisons between lower and higher creativity subgroups are also presented in Online resource (Tables S5 and S6).

### Intervention characteristics

First, we assessed associations between creativity rating and the characteristics of the interventions (Table 2). Creativity rating was not associated with study quality categories (weak/moderate/strong), *F*(2, 90) = 0.97 *p* = 0.38, η^2^ = 0.02 (Table 2). The distributions of creativity scores for interventions with EF or AA measures were similar (Table 2). An independent t-test showed no significant difference in creativity score between studies with EF or AA measures (*t*(92) = 0.6, *p* = 0.53). Studies with an active control group had interventions with lower creativity levels on average than those with a business as usual control group (*t*(91) = 3.3, *p* < 0.001). Studies with a sedentary control group also had lower creativity levels on average than those with a physically active control group (*t*(91) = 4.1, *p* < 0.001, Table 2). Of all the interventions included, none had a frequency of less than once per week (Table 2). The type of intervention determined the frequency per week. For example, physically active breaks from academic work tend to be shorter and can be carried out more often during the school week than longer sessions involving traditional physical activities. The frequency of practice was associated with the creativity rating of the intervention, *F*(3, 89) = 10.42, *p* < 0.001, with a large effect size (η^2^ = 0.26). Pairwise comparisons revealed that the higher the number of sessions per week, the less creative the intervention (pairwise comparisons: 1/week vs. 3/week or 4/week, *p* < 0.001, 2/week vs. 3/week, *p* = 0.02, 2/week vs. 4/week, *p* = 0.03). The duration of interventions ranged between three weeks^29^ and six years^84^. Intervention creativity rating was not associated with intervention duration, *F*(3, 89) = 2.10, *p* = 0.11, η^2^ = 0.07.

**Table 2:** Number of studies and descriptive statistics of creativity scores as a function of outcome measures, quality of study, frequency, session and intervention duration, intensity and teacher qualification.

	*n*	Creativity score		
		*M*	*SD*	Median
*All studies*	92	4.81	3.50	4.17
Lower creativity	60	2.72	2.03	2.17
Higher creativity	32	8.98	1.47	9.00
*Measures*^†^				
Executive function	45	5.01	3.59	4.67
Academic achievement	46	4.59	3.54	3.78
Fluid intelligence	8	4.29	3.98	3.33
On-task behaviour	5	2.80	1.30	3.33
Creativity	5	7.67	3.89	9.00
*Quality of study*				
Weak	10	4.87	2.57	4.67
Moderate	31	5.31	3.95	5.75
Strong	51	4.22	3.30	3.33
*Frequency (number of sessions per week)*				
1	13	8.21	2.04	8.00
2	21	5.97	3.36	6.67
3	27	3.34	3.20	2.00
> 3	31	3.52	2.91	3.00
*Intervention duration*				
≤ 6 weeks	19	3.95	3.41	3.33
7–10 weeks	21	5.38	3.55	5.67
11–24 weeks	28	5.55	3.71	5.67
≥ 25 weeks	24	3.58	2.86	3.41
*Intensity*				
Cannot tell	18	6.63	3.65	6.67
LPA	6	2.22	1.92	2.00
LMPA	2	4.17	4.00	4.16
MPA	20	4.41	3.18	3.33
MVPA	43	4.64	3.46	4.33
VPA	3	2.41	2.10	3.33
*Session duration*				
< 20 min	20	1.30	1.22	1.00
20–44 min	20	4.63	3.45	3.83
45–60 min	42	5.83	3.30	6.25
> 60 min	10	6.99	2.49	7.50
*Teacher qualification*				
Low qualifications	32	3.38	3.18	2.50
High qualifications	60	5.42	3.43	5.00
*Control group type*				
Active	57	3.87	3.41	3.33
Business as usual	35	6.16	3.02	5.92
*Control group physical activity levels*				
Physically active	41	6.10	3.44	6.17
Sedentary	51	3.35	3.02	2.67

*LPA* low physical activity, *LMPA* low to moderate physical activity, *MPA* moderate physical activity, *MVPA* moderate to vigorous physical activity, *VPA* vigorous physical activity.

^†^Some studies contained more than one outcome.

Most of the studies (77%) fell into the MPA or MVPA categories (Table 2). The intensity of practice was not associated with the creativity rating of the PA intervention being tested, *F*(5, 87) = 2.28, *p* = 0.05, η^2^ = 0.12. When considering the duration of each session (i.e., bout), approximately half of the studies fell into 45–60 min (Table 2). Session duration was associated with intervention creativity rating (*F*(3, 69) = 13.67, *p* < 0.001, η^2^ = 0.32). Pairwise comparisons revealed that longer teaching sessions fostered creativity to a greater extent (< 20 min vs. 45–60 min or > 60 min, *p* < 0.001; < 20 min vs. 20–44 min, *p* = 0.01). Finally, teacher qualification was split 50:50 between low and high qualification. Teacher qualification was associated with creativity rating of the intervention, whereby highly qualified educators were more likely to lead interventions with more elements fostering creativity (*t*(91) = 2.83, *p* < 0.01).

In sum, the number of creativity elements used in interventions was higher in studies with lower frequency of practice, longer session duration and higher teacher qualification, but did not associate with intervention duration or intensity of physical activity.

### Creativity elements of physical activity interventions

**Figure 2:**
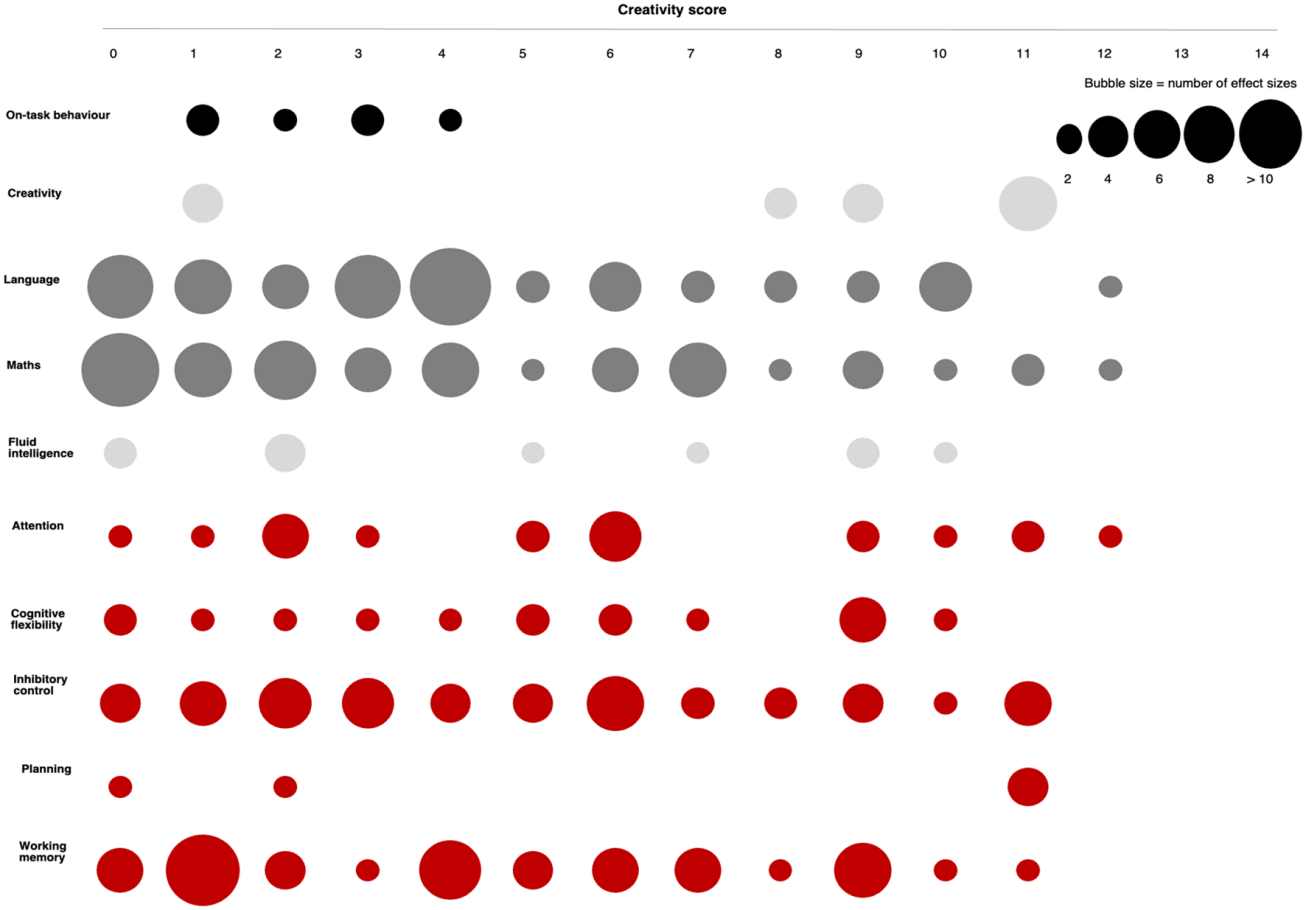
Distribution of creativity score by outcome.

Overall, the interventions were found to vary substantially in the extent to which they fostered creativity. However, there was less variation when comparing studies measuring the same type of outcome. Figure 2 shows the distribution of creativity scores as a function of outcome. There was a range of creativity scores for most outcomes although some had a low number of studies (e.g., planning), and some had a more reduced range of scores (e.g., on-task behaviour).

Studies which measured executive function (*n* = 45) did vary in creativity. Some interventions involved repetitive and minimal social interaction (group/team/opponent) and lacked open-ended instruction (e.g., aerobic or PA with academic instruction interventions incorporating jumping jacks, running on the spot, *n* = 15; yoga, *n* = 3). Others had higher creativity ratings because they incorporated open use of space (dance, *n* = 2), or an opponent (taekwondo, *n* = 2). Highly creative interventions included a combination of varied movements, social interaction and open space (enriched PE, *n* = 10; ball sports, *n* = 4; gymnastics, *n* = 2; creative dance, *n* = 1).

Studies which measured academic performance (*n* = 47) often had interventions which integrated academic instruction with PA (*n* = 22). While most of these interventions (15 out of 22) where low on the creativity scale, incorporating tasks which were repetitive, with minimal social interaction (group/team/opponent), and which lacked open-ended instruction (e.g. jumping jacks, running on the spot, repetitive dance movements or juggling), others were higher on the divergent scale as they supported movement variability through open-ended instructions (creative movement practice, *n* = 1), social interaction (group/team/opponent) or space and equipment (*n* = 6). Within the remaining studies (*n* = 25), some included repetitive movements lacking open-ended instruction and were therefore on the lower end of creativity (skipping, jumping and running, yoga, taekwondo, choreographed dance, *n* = 9), others fostered creativity by adding social interaction (PE increase in dosage or intensity, *n* = 5), while others, higher on the creativity scale, had more varied movements with open-ended instruction and/or social interaction (team ball sports, *n* = 3; enriched PA, *n* = 2; creative dance, *n* = 1).

Studies which measured on-task behaviour (*n* = 5) were low on the creativity scale in general and had similar ratings (Fig. 2). Some interventions were repetitive, with minimal social interaction (group/team/opponent) and lacking open-ended instruction (e.g., PA with academic instruction incorporating aerobic movements such as jumping jacks, running on the spot, *n* = 4). Creativity as an outcome has only started to garner interest in the field of PA research in children. Interventions used in the four of the five studies measuring creativity as an outcome were rated high on the creative scale and incorporated tasks which were variable, with social interaction, open-ended instruction and open space with little use of equipment (creative movement in the form of PE, *n* = 2, dance).

### Associations with creativity rating

**Figure 3:**
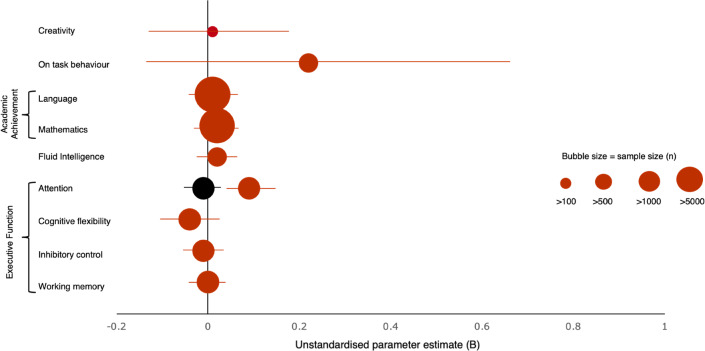
Estimate of the association between effect size and creativity rating of the physical activity interventions. Note: Total effect sizes = 270; total sample size = 25,334 errors bars represent 95% CI. The black indicators reflect effect sizes excluding influential effect sizes.

Three-level meta regression analyses were completed on all outcomes except planning (insufficient number of effect sizes) to test whether the number of elements of the PA interventions fostering creativity positively associated with the effect sizes observed for cognitive or academic performance outcomes (Table 3, Fig. 3). A second set of analyses compared higher and lower creativity intervention groups (Online resource Tables S5 and S6). The results are described below, considering each outcome in turn.

**Table 3:** Moderator analysis of the association between creativity rating of physical activity interventions and effect size for each outcome measure.

	Sample size	*k*	*n* effect sizes	Creativity					Heterogeneity			
				β	*B*	95% CI	*p*	Rating range	*I*^2^ ^level 3^	*I*^2^ ^level 2^	95% CI	Q
Executive functions	8448	45	127	0.06	0.01	− 0.01 to 0.03	0.56	0–12	24.1%	48.9%	66.5 to 72.9%	323.8
Infl. cases removed^1^	7886	45	125	− 0.08	− 0.01	− 0.05 to 0.02	0.53	0–12	81.1%	10.9%	56.2 to 72.1%	766.4
**Attention**	**2999**	**11**	**19**	**0.41**	**0.09**	**0.04** to **0.13**	** < 0.01**	0–12	93.2%	0.0%	77.4 to 93.9%	74.3
* Infl. cases removed*^2^	2847	11	18	− 0.05	− 0.01	− 0.04 to 0.01	0.24	0–12	0.0%	17.6%	0.0 to 17.6%	19.8
Cognitive flexibility	1278	11	16	− 0.35	− 0.04	− 0.09 to 0.02	0.17	0–9	59.9%	0.0%	0.0 to 59.9%	29.1
* Infl. cases removed*^3^	1057	10	15	− 0.36	− 0.04	− 0.09 to 0.01	0.12	0–9	42.9%	0.0%	34.7 to 42.9%	20.9
Inhibitory control	5441	30	41	− 0.09	− 0.01	− 0.10 to 0.04	0.58	0–11	92.8%	0.0%	50.1 to 92.8%	545.7
* Infl. cases removed*^4^	4981	29	40	− 0.44	− 0.07	− 0.24 to 0.11	0.42	0–9	86.4%	12.9%	2.8 to 99.3%	4401.5
Working memory	5287	30	46	− 0.01	0.00	− 0.04 to 0.03	0.96	0–11	7.0%	72.9%	60.9 to 79.9%	148.2
* Infl. cases removed*^5^	5243	29	45	− 0.06	− 0.01	− 0.04 to 0.02	0.59	0–11	6.6%	65.0%	52.5 to 71.6%	123.9
Academic achievement	14,386	47	114	0.08	0.02	− 0.04 to 0.07	0.56	0–12	78.4%	18.7%	75.4 to 97.0%	1898.0
* Infl. cases removed*^6^	13,641	46	113	0.05	0.01	− 0.04 to 0.06	0.71	0–12	78.2%	18.9%	67.2 to 97.0%	1873.9
Language	11,320	32	60	0.02	0.01	− 0.06 to 0.07	0.89	0–12	56.2%	41.4%	73.6 to 97.6%	1066.5
* Infl. cases removed*^7^	11,162	31	59	− 0.05	− 0.01	− 0.08 to 0.06	0.78	1–12	57.2%	40.5%	71.6 to 97.7%	104.01
Maths	13,366	42	54	0.13	0.02	− 0.01 to 0.07	0.43	0–12	83.8%	12.7%	74.2 to 96.5%	829.0
* Infl. cases removed*^8^	13,264	42	53	0.12	0.02	− 0.03 to 0.07	0.42	1–12	96.7%	0.0%	72.5 to 96.7%	821.5
On− task behaviour	804	5	6	0.34	0.22	− 0.16 to 0.79	0.62	1–4	93.7%	3.7%	85.6 to 97.5%	77.5
Fluid intelligence	1351	8	9	0.43	0.02	− 0.03 to 0.07	0.32	0–10	13.7%	0.0%	0.0 to 67.2%	6.9
* Infl. cases removed*^9^	1115	6	7	0.38	0.02	− 0.02 to 0.11	0.45	1–10	0.0%	0.0%	0.0 to 64.8%	3.9
Creativity	345	5	14	0.05	0.01	− 0.16 to 0.18	0.92	1–11	83.2%	1.1%	0.0 to 84.7%	34.6
* Infl. cases removed*^10^	315	5	13	− 0.31	− 0.05	− 0.22 to 0.13	0.55	1–11	84.8%	0.0%	0.0 to 84.8%	26.3

Significant associations are highlighted in bold; k = number of studies; *I*^2^ at study level (level 3: between-study heterogeneity, level 2: within-study heterogeneity); β was estimated based on multiplying *B* by the *SD* of creativity and diving by the *SD* of effect size on the outcome; removed as influential studies: ^1^ ^85,86^; ^2^ ^85^; ^3^ ^87^; ^4^ ^10^; ^5^ ^88^; ^6^ ^89^; ^7^ ^90^; ^8^ ^91^; ^9^ ^92,93^; ^10^ ^94^.

### Executive functions

Combining all studies measuring executive functions as an outcome (*k* = 45) indicated that the extent to which PA interventions fostered creativity did not associate with the interventions’ effect size, with a narrow 95% CI confirming the precision of results (Table 3). Higher vs. lower creativity group analyses showed that there was no difference in effect size between groups and neither group of interventions had a significant impact on EFs (Online resource Table S5). EF sub-domains were also analysed separately.

While a small significant positive association was found between creativity rating and effect size on attention (Table 3), removing an influential effect size had a large impact on the estimated between- and within-study heterogeneity, which dropped to low rates, and the summary effect size became non-significant, suggesting the association with creativity rating was driven by one effect size from a single study (Table 3**, **Fig. 3). This study^85^ had a high creativity rating and was compared to a sedentary control group, while all other highly creative interventions used a physically active control group. Sub-group analysis revealed the same pattern of results (Online resource Table S5).

There was no association between creativity rating of the PA interventions and effect sizes on cognitive flexibility, inhibitory control or working memory measures (Table 3). Group comparisons indicated that the lower creativity group had a small significant positive association for working memory; the summary effect size for the higher creativity group was of similar size but was not significant. The groups did not significantly differ from each other. This was also the case when removing influential effect sizes (Online resource Table S5). There was a significant group differences for cognitive flexibility, indicating that highly creative PA negatively influenced cognitively flexibility. This effect remained the same when removing influential effect sizes (Online resource Table S5). The higher creativity subgroup consisted of three studies and a total of six effect sizes assessing the effect of the Team Games intervention^95^ or two creative dance interventions^29,96^. The two interventions that had a negative effect were taught over a short period (3–6 weeks). In summary, while there was some heterogeneity between studies, the extent to which interventions fostered creativity did not influence their impact on executive functions except for cognitive flexibility.

Four studies included planning measures as outcomes (total of five effect sizes). The intervention implemented by Pesce et al.^97^ involved a cognitively enriched specialist led PA programme and was rated as fostering creativity (rating 11/14). The three other studies used aerobic activities and had lower creativity ratings (0 to 3.5/14)^98–100^. All PA interventions were taught by highly qualified teachers, with all data collected within two weeks of intervention end date. Only one study led to significant improvement in planning performance^99^. This study involved short sessions taught three times per week, with results being measured using a physical implementation of the Tower of Hanoi test of planning in a smaller sample than the other studies. Because of the low number of studies, further analyses testing association with creativity rating or grouping were not carried out.

### Academic achievement

There was no association between creativity rating of the interventions and their effect size on academic achievement overall, or mathematics and language outcomes separately, even after influential studies were excluded (Table 3). Group comparisons indicated that the lower creativity group had a small significant positive association for academic achievement, however the two groups did not significantly differ in mean effect size. This was also the case when removing influential effect sizes (Online resource Table S3). A similar pattern was observed for mathematics outcomes but did not remain significant when the influencing effect size was removed.

### On-task behaviour

There was no significant association between creativity rating and on-task behaviour. The sub-group analysis was not run as all studies were in the lower creativity rating group (see Fig. 2). We identified one influential study^101^, which had smaller sample size than the rest of the studies and used a physically active control group, while the other studies used a sedentary control groups. However, we could not run a sensitivity analysis excluding this study, as there were only four studies with sedentary control groups only.

### Creativity

There was no association between creativity rating of the interventions and effect size on creativity measures (Table 3). However, all but one intervention assessing creativity as an outcome had high creativity ratings (see Fig. 2) and included creative dance or creative physical movement. Sub-group analysis for the highly creative sub-group revealed a large summary effect of this group of interventions on creativity measures (*g* = 0.73, *p* = 0.04 (95% CI 0.06–1.40), *I*^*3*^ = 86.4%, *I*^2^ = 0.0%). Overall, the quality rating of these studies was either moderate or high with only one study having a weak rating for blinding of participants^57^. All but one had highly qualified teachers implementing the intervention with all data collected within two weeks of intervention end date. Regardless of the type of PA, the majority (nine out of 14) of the effect sizes showed participation in these programs had a moderate to large beneficial effect on creativity.

## Discussion

The extent to which physical activity interventions foster creativity was investigated to gain a better understanding of whether this aspect of interventions may influence their impact on cognition and academic achievement in primary school aged children. By considering the performing arts as a type of PA we have attempted to overlap PE and sport literature with the performing arts to understand unifying themes across disciplines^7^ and provide a bridge between physiological and cognitive theories in PA literature^102^. The results indicated that the extent to which the PA interventions may have fostered creativity during the physical activity sessions, whether through the activity practiced, the type of instructions, or the environment, did not positively associate with the size of the effects on measures of executive functions, academic achievement, fluid intelligence, on-task behaviour or creativity. Unexpectedly, more creative PA interventions, as a group (*n* = 3), had a negative impact on cognitive flexibility. While the creativity of ratings of the studies varied for AA and EF outcomes, studies measuring on-task behaviour as an outcome tended to be low in creativity, while studies measuring creativity as an outcome tended to be high in creativity, and positive effects of PA interventions were found for both types of outcomes.

Overall, the creativity of PA interventions did not seem to impact their effects on executive functions. Although we did find attention benefited from higher amounts of creativity, sensitivity analyses indicated this was due to a single effect size in a study incorporating a cognitively enriched activity fostering higher creativity^85^. This intervention was taught by lower qualified teachers twice per week over a 20-week period and had a large positive effect on attention compared to a sedentary control group. In comparison, two other studies measuring the effects of a cognitively enriched activity had a similar intervention duration (20 or 24 weeks) but a frequency of once per week instead of twice per week, and physically active control groups and showed (a) a moderate positive effect on attention (teaching by higher qualified teachers^97^); (b) a small negative effect on attention (teaching by a lower qualified professional^10^). With only four studies it is difficult to make strong conclusions, but these results are in line with the idea that consistency is required to have an effect when it comes to highly creative PA when lower qualified professionals are teaching creative physical activity^103^. Notably, we did not find any effect of creativity on inhibitory control. Previous research has suggested a link between various characteristics of PA (e.g. decision making, tactical cooperation and task variability) and inhibitory control, which was not found in this meta-analysis^65^. On the contrary we did find differences in the effects of lower vs. higher creativity interventions on cognitive flexibility. The higher creativity sub-group showed negative effects on cognitive flexibility. This group included three studies, with six effect sizes between them, leading to a small negative summary effect on cognitive flexibility^29,95,96^. One study involved team games, and was contrasted to PE, the other two included creative dance and were contrasted to choreography dance or a music intervention. The three interventions were shorter (3–10 weeks) than the lower creativity interventions (20–44 weeks except for one study that was 7-weeks long). It could be that interventions that foster creativity have an additional cognitive load that hinders the effects on cognitive flexibility. It has been suggested that non-neurotypically developing children benefit more from PA interventions in the sub-domain of cognitive flexibility^104,105^. More work would be needed to explore this potential negative effect.

Neurobiological studies in animals support the idea that task variability and complexity during motor performance can foster neuroplasticity and skill learning^106^. Researchers have classified these characteristics of PA as cognitively engaging^2,9^. However, grouping together a wide range of different PA types without considering how enriched each aspect makes them, can be misleading. For example, yoga has been classified as cognitively engaging in other meta-analytic reviews^2^, however the practice of it and the instructional context is repetitive, using teacher demonstration and is also lacking in social interaction. Here our aim was to provide evidence that considering whether the characteristics of PA intervention foster creativity is an aspect of cognitive engagement that may be relevant for improving cognitive outcomes. However, as no association was observed for executive function measures, even though overall the PA interventions were found to benefit working memory and fluid intelligence^56^ other characteristics will need to be considered. Notably, aerobic and/or moderate to vigorous intensity interventions were found to benefit EF as a whole in our companion paper, suggesting that EF may be more susceptible to improvements mediated by neurophysiological mechanisms^56^.

Our expectation was that higher level aspects of cognition such as planning and fluid intelligence would be impacted by the extent to which the PA interventions fostered creativity. There were too few studies assessing planning as an outcome to test for an association. No effect of intervention creativity was found for fluid intelligence, which was instead benefitted from PA interventions as a whole^56^. Aspects of a PA task and environment that give the learner a choice have been thought to lead to deep processing^7,107^. We expected that allowing learners greater autonomy would transfer to planning and problem-solving skills. The contextual interference effect occurs in motor learning and varies depending on how PA training is implemented^108^. Variability in physical training “interferes” with the skill being learnt resulting in poorer performance in the practice session but leading to transfer of learning to other tasks^109,110^. For example, in choreographic dance, the dancer will repeat the choreography movement sequence to remember and perform it, whereas in a creative dance session, the moves are created in the moment and are rarely repeated. The freedom of choice given by the instructional conditions (e.g., problem-solving instructions) in a creative physical practice gives the learner time to solve a task and not perfect the same move. This contextual interference gives rise to creative and motor skill demands that do not seem to transfer to greater improvements in fluid intelligence than other types of PA. It may be that beneficial effects would be observed if a more proximal problem-solving or reasoning task was used, e.g. involving body movements.

Four of the five studies assessing creativity as an outcome used interventions rated high on fostering creativity, and no association between effect size and creativity rating was observed across these few studies. Moderate to large positive effects on creativity outcomes were reported in the studies reviewed here, which is promising, however this may reflect publication bias. It has been suggested that holistic physical practices influence planning and creativity outcomes^108^. Specifically, researchers have proposed that the mechanism linking PA to creativity is not a link between exercise and cognition but that the broadening of attention during free movement compared to restricted movements^111,112^. Murali and Händel^111^ conducted three experiments in adults involving sitting and walking to identify the mechanism between movement and creativity through restricting movement in both activities. They found that free movement led to better performance in creativity tasks due to broadening of focus of attention (see also^112^). This provides initial evidence that restricting PA (e.g. through drills, copying) will not improve creativity^111^. Applying the same free movement tasks to children may not be as applicable as a younger population may not have the self-regulatory skills to manage their behaviour and follow the instructions to restrict their movements^113^. Affect may play a role in creative PA interventions. Positive affect is related to producing more original ideas^114,115^, and it has been suggested that PA can reduce stress (negative effect)^116^. To date, studies focussing on children have not investigated this mediating pathway. Future research could start exploring how positive affect and reduced stress may mediate effects of PA on creativity^38,117^.

PA intervention that fostered creativity to a greater extent did not benefit academic achievement. Over half of the studies measuring mathematics performance applied PA with academic instruction; most of these had few characteristics that fostered creativity. The majority of interventions in the higher creativity subgroup used a variety of PA, ranging from sports such as football^118^ and basketball^119^ to cognitively enriched games^93,120,121^. Results of our companion analysis indicated that PA with academic instruction benefited mathematics achievement, suggesting possible near-transfer effects, while enriched PA programmes benefited language achievement^56^.

Studies investigating on-task behaviour mainly trained PA with academic instructions. While these studies showed overall beneficials effects of PA on on-task behaviour^56^, the interventions were on the lower spectrum of creativity ratings and no association with creativity was found. The mechanism of impact of creativity elements of PA intervention on on-task behaviour remained to be investigated with a broader range of PA. One meta-analysis of 24 intervention studies looking at PA and school engagement in children and youths found significant small improvements for school engagement after practicing PA^122^. The researchers suggest that it is the novelty of distraction away from academic tasks which gives space to refocus^123,124^. One possibility is that the added cognitive load and complexity of the tasks, but also possibly how engaging these activities are (e.g. playing in a group rather than alone, or coming up with new strategies rather than repeating drills) may foster motivation and focus during PE classes, and these may then be sustained in other classes.

### Strength and limitations

This meta-analysis intended to build on prior analyses, while specifically focusing on the task and environmental aspects of interventions that foster a creative PA and how these characteristics may influence cognitive and academic outcomes in children. This work extended the evidence by including recent PA interventions that incorporated activities such as dance and creative movement (*n* = 6), which possibly better differentiated the effects of the creative aspects of PA. Although recently published meta-analyses have attempted to understand qualitative aspects (e.g. cognitively engaging PA^2,9^) and their effects on outcomes, this is the first study to tease apart specific aspects (task and environment) of an activity and collectively understand the effect they have on educational outcomes. A strength of the study is the use of multi-level modelling, allowing the inclusion of more than one effect size per study. Two main limitations of the analyses are that we could not control for the extent to which the PA control conditions, including business as usual PE, included elements fostering creativity or could not run sensitivity analysis of the higher/lower creativity group comparison for some outcomes because of the small number of studies. Some types of outcomes such as on-task behaviour and creativity tended to be evaluated for interventions rating low or high on the creativity scale, limiting our analyses. Further research is required to better understand how PA should be structured, particularly exploring different pedagogical practices. In addition, measuring outcomes with instruments that are relevant to the activity or real-world test, in this case using physically active instruments may provide more information regarding near and far transfer effects and possible mechanisms of impact. Other limitations include the limited number of studies for planning, moderate agreement between raters for the creativity rating measure, and the fact that studies with higher creativity interventions were less likely to use an active control group rather than a business-as-usual group, limiting the strength of the evidence.

### Implications for research and practice in education

UNESCO members advocate for quality physical education provision in schools^125^. This includes social and emotional competencies as well as physical competencies. The research field should begin to incorporate these unexplored characteristics of PA in intervention studies. The movement literature has started moving towards this view by incorporating tasks with an emotional component, however this is in its infancy (e.g.^126,127^). Future research could also focus on psychosocial variables, even though this can be challenging when testing younger participants. Movement can be important to succeed in domains outside of physical education, and interventions fostering creative physical activities may foster children’s creativity more broadly. With schools and governments understanding the importance of not reducing the time spent on PE and PA, it is now important to know how it can benefit students in other domains and how best to use that time.

## Conclusion

The primary goal of this review was to identify and highlight whether task and environmental factors that contribute to creative PA may benefit children’s cognition and academic performance. Activities can be taught in different ways by using different instructional methods, for example, which could provide the conditions to enhance cognitive and educational outcomes and thus allow a child to make choices and experience the effect of their own physical actions. By considering individual facets of different “types” of physical activities, our aim was to provide a broader framework of qualitative aspects of PA to continue progress in this field of research. Our results do not show that creative PA broadly benefits academic achievement or executive function, but that creative PA does lead to improvements in measures of creativity. Future intervention studies should consider measuring outcomes requiring body movements, which may reflect near-transfer effect, and/or instruments measuring affect or stress, which may reflect mediating pathways of interest.

## Supplementary Information

Supplementary Information.

## Data Availability

The datasets generated during and/or analysed during the current study are available in the osf repository, https://osf.io/uz258/.
