# Supplementary material for: Multi-level meta-analysis of whether fostering creativity during physical activity interventions increases their impact on cognitive and academic outcomes during childhood
Source: Sci Rep. 2023 May 24;13:8383. doi: 10.1038/s41598-023-35082-y (PMC10209177; doi:10.1038/s41598-023-35082-y)
Supplement: Supplementary file 1 — Supplementary Information. [file 41598_2023_35082_MOESM1_ESM.pdf]

Multi-level meta-analysis of whether fostering creativity during physical activity interventions increases their impact on cognitive and academic outcomes during childhood

Fotini Vasilopoulos[1,2], Holly Jeffrey[3], Yanwen Wu[3], & Iroise Dumontheil[1,2]

[1] Centre for Brain and Cognitive Development, Department of Psychological Sciences, Birkbeck, University of London, London, UK

[2] Centre for Educational Neuroscience, University of London, London, UK

[3] University of Cambridge, Cambridge, UK

Corresponding author:

Fotini Vasilopoulos

Department of Psychological Sciences

Birkbeck, University of London

Malet Street, WC1E 7HX, London, UK

fvasil01@mail.bbk.ac.uk

Table S1.

	Lower creativity	Higher creativity	0	1	2
TASK CHARACTERISTICS	Repetitive	Varied	Block training such as learning a set move in ballet or forehand swing in tennis until it is mastered.	A mixture of the two.	A variety of activities to learn. Using different tennis games with different rules to master a forehand.
	1. Individual<br>2. Parallel<br>3. No opponent	1. Group<br>2. Team<br>3. Opponent	Practicing individually with no interaction with others such an aerobics class.	A mixture of the two.	Interaction with someone whether that be competitively or not.
	Technical acquisition	Real world activity	Drills to master motor skills.	A mixture of the two.	A sport or holistic movement activity practiced as it is e.g. tennis or martial arts.
	Convergent task	Divergent task	Does not allow for exploration and is prescriptive. For example, copying in ballet.	A mixture of the two.	Allows for exploration such as PA games or creative movement.
ENVIRONMENT	Technical instruction and demonstration	Open-ended instruction	Copying the teacher's movements. For example, in a dance choreography or aerobics class.	A mixture of the two.	Open-ended instructions include "*Show me any kind of…*", "*Show me any other way to…*", "*How would you do that differently?*"
	Fixed space	Open space	Court, swim lanes or track.	A mixture of the two.	An indoor or outdoor space with changing boundaries or no boundaries.
	No props	Props	Use of just the body. For example, aerobics class such cross fit.	A mixture of the two	Music in dance or balls in sports.

Table S2. Search Terms

Intervention	Population	Setting	Design	Outcomes
danc*	child*	class*	train*	acad*
gymnas*	NOT ADHD	school	practice	achiev*
physical* active	NOT autis*		curriculum	attain*
activity	NOT obes*		intervention	math*
PE			teach*	numeracy
sport			instruct*	literacy
fitness			class	reading
exercis*			trial	cognit*
creativity creative				executive function
theat*				attention*
				inhibit*
				flexibility
				switch*
				shift*
				plan*
				working memory
				updating

Table S3. Eligibility Criteria

	Inclusion Criteria	Exclusion Criteria
Population	Participants aged between 5 and 12 years old	Participants with a developmental delay, developmental disorder, obesity, physical or mental illness
Intervention	Physical activities (PA), defined as any activity that encourages children to move<br><br>PA programs that were manipulated within the study and included a minimum of three sessions over the course of more than one week.	Single bouts of PA<br><br>PA that is considered elite training<br><br>An activity that is creative but does not include PA (e.g. painting, drawing, music)
Comparison	Active or passive control group	No comparison group
Outcomes	Direct assessment using objective or standardised measures of cognitive outcomes (working memory, inhibitory control, cognitive flexibility, planning, attention, fluid intelligence, creativity or on-task behaviour) or academic achievement (mathematics or language)	Subjective measures such as surveys, questionnaires, or interviews that are self-report or completed by teachers or parents<br><br>Objective measures of other aspects of cognition (e.g. IQ)
Study Design	Randomised controlled trials (RCTs), pilot-, cluster-, or quasi-RCTs, controlled before and after studies	Observational studies (e.g., cross-sectional, cohort, case-control), quasi-experimental studies, non-randomised control trials, or case studies
Publication	Peer-reviewed<br>Written in English	Reviews, editorials, policy reviews, statements, or commentaries

Table S4. Study Characteristics

Study	n	M age	% Female	Physical activity intervention	Duration (weeks)	Freq. (per week)	Intensity	Session duration (min)	Teacher qualification	Control group(s)	Quality	Outcomes	Instrument
Aadland et al. 2019	1202	10.2	-	Active Smarter Kids (PA with academic instruction)	20	3	MVPA	30	Classroom teacher (LQ)	PE	Strong	Language<br>Maths	Standardised national test
Ahamed et al. 2007	287	10.2	50.2%	AS! BC (PA break from academic instruction)	50	5	MPA	15	Classroom teacher (LQ)	PE	Strong	AA composite	Canadian Achievement Test (CAT-3)
Barnard et al. 2014	149	7.4	53.0%	Integrated PA (PA with academic instruction)	8	3	LPA	30	Not reported (LQ)	1. Intensive PA 2. Passive	Weak	Language<br>Maths	ESSI Reading<br>ESSI Spelling<br>Vassi Mathematical Skills
Beck et al. 2016	165	7.5	46.7%	Gross Motor Math (PA with academic instruction)	6	3	LMPA	60	Classroom teacher (LQ)	1. Fine Motor Math (not PA) 2. Academic Instruction	Strong	Inhibitory control<br>Maths<br>Working memory	Eriksen Flanker Task<br>Standardised test<br>CANTAB: Spatial Span subset
Beckmann et al. 2022	481	8.1	49.0%	KazKidz	12	2	Cannot tell		HQ	Placebo	Strong	Inhibitory control<br>Language<br>Maths	Eriksen Flanker Task<br>School Performance Test
Bugge et al. 2018	1181	8.4	53.3%	PE Boost	234	4	cannot tell	45	PE teachers (HQ)	PE	Moderate	Language<br>Maths	Danish National Test System
Bunketorp et al. 2015	545	-	46.4%	School-in-Motion: different sports and games with or without use of equipment	8	2	MVPA	30	Experienced exercise instructor (HQ)	PE	Moderate	Language<br>Maths	Standardised national test
Cecchini & Carriedo 2020	46	6.4	58.7%	math-PE integrated teaching	3	5	MVPA	60	PE teachers (HQ)	PE	Strong	Maths	Standardised test
Chaddock-Heyman et al. 2013	23	8.9	56.5%	Fitness Improves Thinking in Kids (FIT Kids) (Aerobic)	30	5	MVPA	120	University staff (HQ)	Passive	Weak	Inhibitory control	Eriksen Flanker Task
Chaddock-Heyman et al. 2020	150	8.5	54.3%	Fitness Improves Thinking in Kids (FIT Kids) (Aerobic)	30	5	MVPA	120	University staff (HQ)	Passive	Strong	Fluid intelligence<br>Language<br>Maths	Kaufman Test of Educational Achievement<br>WJ III: Concept Formation, Planning, Verbal Comprehension, Visual Auditory Learning

Study	n	M age	% Female	Physical activity intervention	Duration (weeks)	Freq. (per week)	Intensity	Session duration (min)	Teacher qualification	Control group(s)	Quality	Outcomes	Instrument
Chaya et al. 2012	193	7.7	49.7%	Yoga	12	6	LPA	45	Experienced yoga teacher (HQ)	PA - passive stretching and aerobic exercises (running and group games)	Strong	Fluid intelligence<br>Language<br>Maths	Malinas Intelligence Scale for Indian Children (MISIC). Indian adaptation of the WISC-II.
Cichy et al. 2020	47	7	44.7%	PE with Eduball	39	2	cannot tell	45	Classroom teacher (LQ)	PE	Moderate	Maths	Standardised national test
Coe et al. 2006	214	11.5	49.0%	PE	20	5	cannot tell	55	Not reported (LQ)	Alternative exploratory class (i.e., arts and computer classes)	Weak	AA composite	Standardised national test
Condello et al. 2021	181	10.7	50.0%	Games-based Multisport		1	Cannot tell		HQ	PE	Moderate	Inhibitory control<br>Working memory	Random Number Generation Task
Crova et al. 2014	70	9.6	50.0%	Tennis skill-based training	21	1	MVPA	120	PE teachers (HQ)	PE	Weak	Inhibitory control<br>Working memory	Random Number Generation Task
D'Souza & Wiseheart 2018	75	7.8	58.7%	Dance: Creative Movement	3	5	MPA	120	Experienced dance teacher (HQ)	1. Music<br>2. Passive	Strong	Cognitive flexibility<br>Fluid intelligence<br>Inhibitory control<br>Language<br>Working memory	Color-shape switch task<br>Digit Span Backward<br>Eriksen Flanker Task<br>K-BIT (Kaufman brief Intelligence Test) matrices<br>PPVT (Peabody Picture Vocabulary Test)<br>Quantity Identity Task<br>Self Ordering Point Task<br>Stroop Colour Test
Dalziell et al. 2015	46	9.5	52.2%	Better Movers and Thinkers (BMT) Enriched PA programme	16	2	cannot tell	60	PE teachers (HQ)	PE	Moderate	Fluid intelligence<br>Language<br>Working memory	The Lucid Assessment System for Schools: phonological composite, reading composite, reasoning, spelling, visual spatial memory subtest.
Dalziell et al. 2019	150	10.5	cannot tell	Better Movers and Thinkers (BMT)	16	2	cannot tell	60	PE teachers (HQ)	PE	Strong	EF composite	Cognitive Assessment System

Study	n	M age	% Female	Physical activity intervention	Duration (weeks)	Freq. (per week)	Intensity	Session duration (min)	Teacher qualification	Control group(s)	Quality	Outcomes	Instrument
				Enriched PA programme									
DeBruijn et al. 2020	891	9.2	51.2%	Aerobic intervention	14	4	MVPA	30	PE teachers (HQ)	1. Cognitively engaging activity<br>2. PE	Strong	Language<br>Maths	Standardised tests
DeGreeff et al. 2016	499	8.1	54.7%	F&V (PA with academic instruction)	44	3	MVPA	20	Classroom teacher (LQ)	Passive	Moderate	Cognitive flexibility<br>Inhibitory control<br>Working memory	Digit Span Backward<br>Stroop Colour Test<br>Visual Span Backward<br>Wisconsin Card Sorting Test
Donnelly et al. 2017	584	8.1	51.4%	Physical Activity Across the Curriculum (PAAC)	117	10	MVPA	10	Classroom teacher (LQ)	Passive	Strong	Language<br>Maths	Wechsler Individual Achievement Test-Third Edition
Egger et al. 2019	142	7.9	54.9%	PA breaks (high cognitive engagement, high physical exertion) (PA break from academic instruction)	20	10	MVPA	10	Classroom teacher (LQ)	1. Aerobic group<br>2. Cognitive group (low physical exertion)	Strong	cognitive flexibility<br>Inhibitory control<br>Language<br>Maths<br>Working memory	Backwards Colour Recall task<br>Eriksen Flanker Task<br>Hamburger Schreib-Probe: Spelling<br>Heidelberger Rechentest: Maths<br>Salzburger Lesescreening: Reading
Fedewa & Davis, 2015	460	grade 3 -5	cannot tell	Classroom-based physical activity breaks	25	20	MPA	5	Classroom teacher (LQ)	Passive	Strong	Fluid intelligence<br>Language<br>Maths	Standard Progressive Matrices<br>National standardised tests
Fedewa et al. 2018	460	grade 3 -5	51.0%	Academic-based movement breaks	36	10	LPA	10	Video (HQ)	Aerobic-only movement breaks	Strong	Language<br>Maths	FastBridge: Standardised tests
Fisher et al. 2011	64	6.1	55.0%	Aerobic PE curriculum	10	2	MVPA	60	Researchers, PE and Classroom teachers (HQ)	PE	Moderate	Attention<br>Fluid intelligence<br>Planning<br>Working memory	CANTAB: attention and working memory subset<br>CAS: planning and simulteanous scale subset
Frischen et al. 2019	76	5.7	60.1%	Yoga	20	3	LPA	20	Researchers (HQ)	1. Rhythm training<br>2. Pitch training	Moderate	Cognitive flexibility<br>Inhibitory control<br>Working memory	Corsi Block Test<br>Dimensional Change Card Sort<br>Matrix Span Test<br>NEPSY-II "statue" subtest

Study	n	M age	% Female	Physical activity intervention	Duration (weeks)	Freq. (per week)	Intensity	Session duration (min)	Teacher qualification	Control group(s)	Quality	Outcomes	Instrument
Fernandes et al. 2022	47	10.5	50.0%	Capoeira		3	Cannot tell	60	HQ	Passive	Strong	Working memory<br>Inhibitory control<br>Maths<br>Language	WISC IV Digit span backward<br>WISC IV Letter number sequencing
Gall et al. 2018	663	9.2	51.0%	Moving to Music	20	1	MPA	45	Classroom teacher and Researchers (HQ)	PE	Strong	Attention<br>AA composite	d2 Test of Attention<br>End of year results
Gallotta et al. 2015	230	9.5	cannot tell	Coordinative PA	20	2	MVPA	60	Classroom teacher (LQ)	1. Traditional PA<br>2. Passive	Moderate	Attention	d2 Test of Attention
Garcia-a-Hermoso et al. 2020	170	10.1	43.5%	Active Start (PE boost)	8	5	MVPA	30	Sport Science Graduate (LQ)	PE	Strong	Attention<br>Language<br>Maths	d2 Test of Attention<br>End of year results
Giordano & Alesi 2022	75	5.7	48.0%	Enriched programme		3	Cannot tell	10	HQ	Free play<br>Passive	Moderate	Inhibitory control	Stroop Test<br>Head Shoulders Knees Toes
Have et al. 2018	505	7.2	50.5%	Active Math (PA with academic instruction)	30	6	MVPA	45	Classroom teacher (LQ)	Academic instruction	Moderate	Inhibitory control<br>Maths	Eriksen Flanker Task<br>Standardised test
Hillman et al. 2014	221	8.8	46.2%	Fitness Improves Thinking in Kids (FIT Kids)<br>(Aerobic)	30	5	MVPA	120	University staff (HQ)	Passive	Strong	Cognitive flexibility<br>Inhibitory control	Color-shape switch task<br>Eriksen Flanker Task
Hraste et al. 2018	36	10.4	cannot tell	Integrated lessons of mathematics/geometry and PA	4	4	LMPA	45	Classroom teacher (LQ)	Academic instruction	Moderate	AA composite	National standardised test
Hsieh et al. 2017	44	8.6	47.7%	Gymnastics	8	2	MPA	90	PE teachers (HQ)	Passive	Weak	Working memory	Delayed matching-to-sample test
Jia et al. 2021	40	6.1	50.0%	Diverse exercise		3	MPA		HQ	Aerobic	Moderate	Language<br>Maths	Wechsler Individual Achievement Test-Third Edition
Kamijo et al. 2011	36	9.0	52.8%	After-School PA Intervention (aerobic)	30	5	MVPA	120	University staff (HQ)	Passive	Moderate	Working memory	Sternberg task
Koutsandreou et al. 2015	71	9.4	54.9%	Cardiovascular Fitness Training	10	3	MVPA	45	Experienced exercise instructor (HQ)	1. Motor skills training<br>2. Academic instruction	Strong	Working memory	Letter Digit Span

Study	n	M age	% Female	Physical activity intervention	Duration (weeks)	Freq. (per week)	Intensity	Session duration (min)	Teacher qualification	Control group(s)	Quality	Outcomes	Instrument
Kvalo et al. 2017	449	9.5	48.7%	Active School (PA with academic instruction)	38	2	MPA	45	Classroom teacher (LQ)	PE	Strong	Inhibitory control<br>Working memory	Backward digit span task<br>Stroop Golden interference effect
Lakes & Hoyt 2004	193	cannot tell	51.3%	Taekwondo	12	2	MVPA	45	Experienced martial arts instructor (HQ)	PE	Strong	Maths<br>On-task behaviour<br>Working memory	Cognitive subscale of Response to Challenge Scale<br>WISC III-FD Arithmetic and Digit Span
Lakes et al. 2013	59	12.2	52.0%	Taekwondo	36	2	VPA	45	Experienced martial arts instructor (HQ)	PE	Strong	EF composite<br>Inhibitory control	The Hearts & Flowers test
Leandro et al. 2018	117	7.5	43.6%	Creative dance & mathematics<br>(PA with academic instruction)	5	1	MPA	60	Classroom teacher (LQ)	Academic instruction	Moderate	Maths	Standardised test
Lin et al. 2021	50	8.6	48.0%	Gymnastics	8	2	MPA	90	PE teachers (HQ)	Passive	Strong	Working memory	Spatial working memory delayed-matching test
Lind et al. 2018	931	11.9	51.0%	Football	11	2	MVPA	45	Classroom teacher (LQ)	PE	Strong	Working memory	Cogstate® Brief Battery: one back (OBK)
Marson et al. 2021	30	8.5	30.0%	Quadrato Motor Training (QMT)		4	Cannot tell		HQ	One Minute Meditation	Weak	Creativity	Alternative Uses Test
Mavilidi et al. 2018	55	10.3	47.3%	Thinking While Moving in English (TWM-E)<br>(PA with academic instruction)	4	3	MPA	40	Researchers, PE teacher (HQ)	Passive	Weak	Inhibitory control<br>Language<br>On-task behaviour<br>Working memory	Eriksen Flanker Task<br>Grammar and Punctuation test<br>Momentary time sampling<br>n-back task<br>National standardised test
Mavilidi et al. 2020	283	9.8	48.4%	Thinking While Moving in English (TWM-E)<br>(PA with academic instruction)	6	3	MPA	40	Classroom teacher (LQ)	Passive	Strong	Language<br>On-task behaviour	National standardised test<br>Momentary time sampling
Mavilidi & Vazou 2021	560	10	51.0%	Move for thought		3	Cannot tell		HQ	Academic	Moderate	Maths	Academic Achievement Test
Mazzoli et al. 2021	141	7.7	46.1%	Cognitively engaging Active Breaks		4	Cannot tell		HQ	Academic instruction	Moderate	Attention<br>Inhibitory control<br>Working memory	Go No/Go<br>List sorting working memory test (NIH Toolbox)

Study	n	M age	% Female	Physical activity intervention	Duration (weeks)	Freq. (per week)	Intensity	Session duration (min)	Teacher qualification	Control group(s)	Quality	Outcomes	Instrument
McMahon et al. 2003	721	6.5	cannot tell	Basic Reading Through Dance (PA with academic instruction)	10	2	MPA	40	Experienced dance teacher (HQ)	Passive	Strong	Language	PhonoGraphix Standardised Tests
Mead et al. 2016	81	11.5	51.9%	Activity breaks from academic instruction	40	5	MVPA	10	Classroom teacher (LQ)	1. Stability Balls<br>2. Passive	Strong	Maths	National standardised test<br>Measure of academic progress test
Meijer et al. 2021	650	9.17	50.6%	Cognitively engaging	14	4	MVPA		HQ	Passive	Weak	Attention<br>Working memory<br>Inhibitory control	Attention Network Task<br>WISC III-FD Digit span<br>Stop and Signal Task
Meloni & Fanari 2019	85	9.3	51.8%	Basketball	30	1	MVPA	60	Not reported (LQ)	Chess	Strong	Language<br>Maths	Standard Progressive Battery<br>Studying test
Morris et al. 2022	97	9.6	52.6%	Maths on the move		1	MVPA		HQ	Academic	Moderate	Maths	
Moreau et al. 2017	318	9.9	61.3%	HIT (High Intensity Training)	6	5	VPA	10	Video (HQ)	Sedentary Games	Strong	Inhibitory control<br>Working memory	Backward digit span task<br>Stroop Test<br>Eriksen Flanker Task
Mullender-Wijnsma et al. 2015	228	8.1	46.5%	F&V (PA with academic instruction)	21	3	MVPA	10	Classroom teacher (LQ)	Passive	Moderate	Language<br>Maths	One-Minute Test - Reading<br>Speed Test Arithmetic
Mullender-Wijnsma et al. 2016	499	8.1	45.2%	F&V (PA with academic instruction)	44	3	MVPA	20	Classroom teacher (LQ)	Passive	Strong	Language<br>Maths	One-Minute Test - Reading<br>Speed Test Arithmetic<br>Child Academic Monitoring System - Maths & Spelling
Mullender-Wijnsma et al. 2019	499	8.1	54.7%	F&V (PA with academic instruction)	44	3	MVPA	20	Classroom teacher (LQ)	Passive	Strong	Language<br>Maths	One-Minute Test - Reading<br>Speed Test Arithmetic<br>Child Academic Monitoring System - Maths & Spelling
Nobre et al. 2022	211		50.0%	Mastery climate		3	Cannot tell		HQ	Traditional PA	Moderate	Maths<br>Language	
Neville & Makopoulou 2021	40	7.4	50.0%	Dance-based PE	6	1	MPA	50	PE Graduate (LQ)	PE	Moderate	Creativity	Guilford's (1967) Alternative Uses task
Norris et al. 2018	219	8.6	49.3%	Virtual Traveller	6	3	MVPA	10	Classroom teacher (LQ)	Passive	Strong	On-task behaviour	Observing Teachers and Pupils in Classrooms (OPTIC) tool
Oppici et al. 2020	80	8.8	61.0%	Dance (high cognitive)	7	2	MPA	60	Experienced dance teacher (HQ)	1. Dance (low cognitive)<br>2. PE	Strong	Cognitive flexibility<br>Inhibitory control<br>Working memory	Dimensional Change Card Sort<br>Eriksen Flanker Task<br>List sorting working memory test (NIH Toolbox)

Study	n	M age	% Female	Physical activity intervention	Duration (weeks)	Freq. (per week)	Intensity	Session duration (min)	Teacher qualification	Control group(s)	Quality	Outcomes	Instrument
Pesce et al. 2013	250	6.9	49.2%	Cognitively Enriched Specialist Led PA	20	1	Cannot tell	60	PE teachers (HQ)	1. Specialist Led PE<br>2. PE	Strong	Attention<br>Planning	CAS: attention and planning sub-scale
Pesce et al. 2016	460	7.5	50.0%	Enriched PE (high cognitive)	24	1	Cannot tell	60	PE teachers (HQ)	PE	Moderate	Attention<br>Inhibitory control<br>Working memory	CAS: attention sub-scale<br>Random Number Generation Task
Pesce et al. 2021	116	8.8	53.0%	Self control programme		1	Cannot tell		PE teachers (HQ)	PE	Moderate	Inhibitory control<br>Attention	
Reed et al. 2010	155	10	43.2%	PA fundamental skills in Core Curriculum (PA with academic instruction)	12	3	MPA	30	Researchers and Classroom teachers (HQ)	Academic instruction	Moderate	Fluid intelligence	Standard Progressive Matrices
Resaland et al. 2016	1202	10.2	cannot tell	Active Smarter Kids (PA with academic instruction)	28	3	MVPA	30	Classroom teacher (LQ)	PE	Strong	Language<br>Maths	National standardised tests
Richard et al. 2018	173	9.6	cannot tell	Creative Exercise Program	10	1	MPA	30	PE teachers (HQ)	Conventio nal Exercise Program	Strong	Creativity	Bertsch test motor creativity<br>Runco Creative Assessment Battery
Riley et al. 2015	54	10.5	48.2%	EASY Minds (PA with academic instruction)	6	3	MVPA	60	Classroom teacher (LQ)	Academic instruction	Strong	On-task behaviour	Momentary time sampling
Riley et al. 2016	240	11.1	40.7%	EASY Minds (PA with academic instruction)	6	3	MPA	60	Classroom teacher (LQ)	Passive	Strong	Maths<br>On-task behaviour	Momentary time sampling<br>Standardised test
Rudd et al. 2021	55	6.6	47.0%	Creative dance	10	2	Cannot tell	60	HQ	Choreogra phy dance	Strong	Cognitive flexibility<br>Inhibitory control<br>Working memory	Dimensional Change Card Sort<br>Eriksen Flanker Task<br>List sorting working memory test (NIH Toolbox)
Sanchez-Lopez et al. 2019	1608	5.8	56.3%	MOVI-KIDS (recreational non-competitive physical activity)	26	3	MVPA	60	Experienced exercise instructor (HQ)	PE	Strong	Fluid intelligence<br>Maths	Battery of general and Differential Aptitudes - logic reasoning and numerical factor subsets
Santos et al. 2017	50	9.2	cannot tell	Skills4Genius	20	3	MVPA	60	PE teachers (HQ)	PE (Additiona l)	Moderate	Creativity	Creativity Behavior Assessment in Team Sports<br>Torrance Test of Creative Thinking
Schmidt et al. 2015	181	11.4	54.9%	Team Games	6	2	MVPA	45	PE teachers (HQ)	1. Aerobic Exercise	Moderate	Cognitive flexibility	Eriksen Flanker task<br>n-back task with pictures of fruits

Study	n	M age	% Female	Physical activity intervention	Duration (weeks)	Freq. (per week)	Intensity	Session duration (min)	Teacher qualification	Control group(s)	Quality	Outcomes	Instrument
										2. PE		Inhibitory control<br>Working memory	
Sjowall 2017	470	9.5	48.5%	PE Boost	78	5	VPA	60	Activity leader (LQ)	PE	Weak	Maths<br>Working memory	Arithmetic test<br>Follow instructions task<br>Odd One Out task
Tanir & Erkut 2018	56	9.2	50.0%	Rhythmic Basketball	6	2	MVPA	60	PE teachers (HQ)	Basketball	Weak	Inhibitory control	Stroop Colour Test
Telford et al. 2012	620	7.5	48.8%	Specialist Taught PA	75	2	MVPA	45	PE teachers (HQ)	PE	Moderate	Language<br>Maths	National standardised tests
Telles et al. 2013	98	10.5	38.7%	Yoga	12	5	LPA	45	Experienced yoga teacher (HQ)	PA (Additional)	Strong	Inhibitory control	Stroop Colour Test
Tocci et al. 2022	95	7.7	50.0%	Specialist led enriched programme		1	Cannot tell		Specialist teacher (HQ)		Moderate	Creativity<br>Inhibitory control<br>Working memory	Bertsch test motor creativity<br>Random Number Generation Task
Tottori 2019	58	10.2	42.3%	HIT (High Intensity Training)	4	3	MVPA	10	University staff (HQ)	Passive	Moderate	Planning<br>Working memory	Digit Span Backward (WISC)<br>Tower of Hanoi (5 disk) time
Tseng et al. 2021	55	11.3	51.0%	FIFA11		5	Cannot tell		HQ	Passive	Strong	Attention	Attention Scale
Van den Berg et al. 2019	512	10.9	46.5%	Just Dance	9	5	MVPA	10	Video (HQ)	Academic instruction	Strong	Attention<br>Inhibitory control	Attention Network<br>d2 Test of Attention<br>Stroop Colour test
Van den Berg et al. 2019b	323	11	48.0%	Juggling with Maths	5	4	LPA	5	Video (HQ)	Academic instruction	Strong	Maths	Multiplication tables test
Van der Niet et al. 2016	112	8.8	50.0%	PA Boost (aerobic and cognitive enriched)	22	2	MVPA	30	PE teachers (HQ)	Passive	Moderate	Cognitive flexibility<br>Inhibitory control<br>Working memory	Digit Span Test from Weschler Memory Scale<br>Golden Stroop Colour Test<br>Tower of London<br>Trail-Making Test<br>Visual Memory Span test from Wechler Memory Scale
Van Klaveren & De Witte 2015	178	10.5	36.0%	Football	10	1	MVPA	180	Experienced exercise instructor (HQ)	Passive	Moderate	Language<br>Maths	Test construction by CITO<br>National standardised test
Vazou & Skrade 2017	284	10.0	46.5%	Move for Thought (PA with academic instruction)	8	3	MPA	10	Classroom teacher (LQ)	Academic instruction	Moderate	Maths	National standardised test

Study	n	M age	% Female	Physical activity intervention	Duration (weeks)	Freq. (per week)	Intensity	Session duration (min)	Teacher qualification	Control group(s)	Quality	Outcomes	Instrument
Vetter et al. 2020	170	8.4	52.0%	Playground	6	3	MVPA	30	Classroom teacher (LQ)	Academic instruction	Strong	Maths	NAPLAN test<br>Teacher-designed multiplication-tables test
Watson et al. 2019	341	9.1	52.8%	ACTI-BREAK: a variety of elements including: drama, games, following instructions, and technology	6	15	MPA	5	Classroom teacher (LQ)	Academic instruction	Strong	Language<br>Maths<br>On-task behaviour	Wheldall Assessment of Reading Passages Test<br>Westwood One Minute Tests of Basic Number Facts<br>Adaptation from the Direct Behaviour Rating Scale
Zinelabidine et al. 2022	44	10.3	49.0%	Aerobic dance		2	MVPA		HQ	Passive	Strong	Cognitive flexibility<br>Inhibitory control<br>Working memory	Trail-making test<br>Stroop Test<br>Digit Recall Test

Table S5. Results of analyses comparing higher and lower creativity physical intervention groups' effect on executive function measures

Outcome measure	Creativity rating group	Sample size	*k*	ES	Creativity grouping				Heterogeneity			
					Summary effect size *g*	95% CI	$p_{subgroup}$	$p_{comparison}$	I^2 level 3	I^2 level 2	95% CI	*Q*
Executive function		8448	45	127				0.19	25.7%	47.1%	67.0% - 72.8%	320.7
	Lower	5660	29	88	0.08	-0.02 – 0.19	0.12					
	Higher	2788	16	39	0.12	-0.25 – 0.49	0.53					
Infl. cases removed		7886	45	125				0.97	81.6%	10.6%	57.2% - 92.2%	775.5
	Lower[1]	5250	29	87	0.08	-0.02 – 0.19	0.11					
	Higher[2]	2636	16	38	0.05	-0.31 – 0.41	0.78					
Attention		2999	11	19				**<0.01**	89.7%	1.9%	77.0% - 91.6%	73.0
	Lower	2212	7	13	0.03	-0.03 – 0.10	0.28					
	Higher	787	4	6	0.25	-0.36 – 0.86	0.34					
Infl. cases removed		2847	10	18				0.30	0.0%	18.6%	0.0% - 18.6%	20.1
	Lower	2212	7	13	0.03	-0.03 – 0.10	0.28					
	Higher[2]	635	3	5	0.01	-0.30 – 0.31	0.10					
Cognitive flexibility		1278	11	16				**0.03**	38.9%	0.0%	0.0% - 38.9%	22.4
	Lower	1173	8	10	0.13	-0.10 – 0.35	0.21					
	**Higher**	**105**	**3**	**6**	**-0.27**	**-0.54 – -0.01**	**0.04**					
Infl. cases removed		1057	10	15				**0.04**	26.8%	0.0%	0.0% - 26.8%	5.3
	Lower[3]	952	7	9	0.08	-0.14 – 0.31	0.42					
	**Higher**	**105**	**3**	**6**	**-0.27**	**-0.54 – -0.01**	**0.04**					
Inhibitory control		5441	30	41				0.66	92.9%	0.0%	50.4% - 92.9%	548.7
	Lower	3829	20	29	-0.02	-0.16 – 0.11	0.71					
	Higher	1612	10	12	-0.12	-0.72 – 0.46	0.64					
Infl. cases removed		4981	29	40				0.42	92.0%	0.0%	11.9% - 92.0%	386.8
	Lower	3829	20	29	-0.02	-0.15 – 0.10	0.71					
	Higher[4]	1152	9	11	-0.23	-0.85 – 0.39	0.43					
Working memory		5287	30	46				0.59	0.1%	78.9%	60.7% - 79.6%	145.4
	**Lower**	**3241**	**18**	**31**	**0.16**	**0.02 – 0.29**	**0.02**					
	Higher	2046	12	15	0.22	-0.01 – 0.44	0.05					
Infl. cases removed		5243	29	45				0.98	5.6%	66.0%	52.9% - 71.6%	122.8
	**Lower**	**3241**	**18**	**31**	**0.16**	**0.02 – 0.29**	**0.02**					
	Higher[5]	2002	11	14	0.13	-0.01 – 0.34	0.28					

Note: significant associations are highlighted in bold, *k* = number of studies, I^2at study level, ES = effect size. Removed as influential studies: [1] Kvalo et al., 2017; [2]Gallotta et al., 2015, one ES);[3] Hillman et al., 2014; [4] Pesce et al., 2016; [5] Hsieh et al., 2017.

Table S6. Results of analyses comparing higher and lower creativity physical intervention groups' effect academic achievement measures

					Creativity grouping				Heterogeneity			
**Outcome measure**	**Creativity rating group**	**Sample size**	***k***	**ES**	**Summary effect size *g***	**95% CI**	$p_{subgroup}$	$p_{comparison}$	I^2 level 3	I^2 level 2	**95% CI**	**Q**
Academic achievement		14386	47	114				0.65	78.4%	18.6%	74.0% - 97.0%	1900.2
	Lower	8565	28	75	**0.10**	**0.02 – 0.18**	**0.01**					
	Higher	5821	19	39	0.20	-0.29 – 0.69	0.41					
Infl. cases removed		13641	46	113				0.91	78.2%	18.8%	64.3% - 97.0%	1878.7
	Lower	8565	28	75	**0.10**	**0.02 – 0.18**	**0.01**					
	Higher[1]	5076	18	38	0.15	-0.51 – 0.81	0.65					
Language		11320	32	60				0.96	56.9%	40.8%	73.0% - 97.7%	1066.2
	Lower	6035	18	38	0.07	-0.04 – 0.17	0.20					
	Higher	5285	14	22	0.10	-0.46 – 0.67	0.71					
Infl. cases removed		11162	31	59				0.55	57.2%	40.4%	62.1% - 97.7%	1041.3
	Lower	6035	18	38	0.07	-0.04 – 0.17	0.20					
	Higher[2]	5127	13	21	0.04	-0.57 – 0.65	0.89					
Maths		13366	42	54				0.48	84.1%	12.41%	71.2% - 96.5%	834.7
	Lower	7603	25	37	**0.10**	**0.01 – 0.18**	**0.03**					
	Higher	5733	17	17	0.25	-0.30 – 0.80	0.34					
Infl. cases removed		13264	42	53				0.42	0.0%	96.7%	64.3% - 96.7%	825.8
	Lower[3]	7531	25	36	0.08	-0.02 – 0.18	0.11					
	Higher	5733	17	17	0.25	-0.30 – 0.80	0.34					

Note: Significant associations are highlighted in bold, k = number of studies, I^2at study level, ES = effect size. Removed as influential studies: [1] Leandro et al., 2018; [2] Garcia-a-Hermoso et al., 2020; [3] Mullender-Wijnsma et al., 2015, one effect size.

References

Aadland, K. N., Ommundsen, Y., Anderssen, S. A., Brønnick, K. S., Moe, V. F., Resaland, G. K., Skrede, T., Stavnsbo, M., & Aadland, E. (2019). Effects of the Active Smarter Kids (ASK) physical activity school-based intervention on executive functions: A cluster-randomized controlled trial. *Scandinavian Journal of Educational Research*, *63*(2), 214–228. https://doi.org/10.1080/00313831.2017.1336477

Ahamed, Y., Macdonald, H., Reed, K., Naylor, P.-J., Liu-Ambrose, T., & McKay, H. (2007). School-based physical activity does not compromise children's academic performance. *Med Sci Sports Exerc*, *39*(2), 371–376. https://doi.org/10.1249/01.mss.0000241654.45500.8e

Barnard, M., Van Deventer, K., & Oswald, M. (2014). The role of active teaching programmes in academic skills enhancement of grade 2 learners in the Stellenbosch region. *South African Journal for Research in Sport, Physical Education & Recreation*. https://www.google.com/search?q=Barnard+M%2C+Van+Deventer+KJ%2C+Oswald+MM.+The+role+of+active+teaching+programmes+in+academic+skills+enhancement+of+grade+2+learners+in+the+Stellenbosch+region.+South+African+Journal+for+Research+in+Sport%2C+Physical+Educat

Beck, M. M., Lind, R. R., Geertsen, S. S., Ritz, C., Lundbye-Jensen, J., & Wienecke, J. (2016). Motor-enriched learning activities can improve mathematical performance in preadolescent children. *Frontiers in Human Neuroscience*, *10*. https://doi.org/10.3389/fnhum.2016.00645

Beckmann, J., Nqweniso, S., Ludyga, S., du Randt, R., Gresse, A., Long, K. Z., Nienaber, M., Seelig, H., Pühse, U., Steinmann, P., Utzinger, J., Walter, C., Gerber, M., & Lang, C. (2022). Evaluation of a Physical Activity and Multi-Micronutrient Intervention on Cognitive and Academic Performance in South African Primary Schoolchildren. *Nutrients*, *14*(13). https://doi.org/10.3390/nu14132609

Bugge, A., Möller, S., Tarp, J., Hillman, C., Lima, R., Gejl, A., Klakk, A., & Wedderkopp, N. (2018). Influence of a 2- to 6-year physical education intervention on scholastic performance: The CHAMPS study-DK. *Scand J Med Sci Sports*, *28*(1), 228–236. https://doi.org/10.1111/sms.12902

Bunketorp, K. L., Malmgren H, Olsson E, Lindén T, & Nilsson M. (2015). Effects of a Curricular Physical Activity Intervention on Children's School Performance, Wellness, and Brain Development. *J Sch Health*, *85*(10), 704–713. https://doi.org/10.1111/josh.12303

Cecchini, J. A., & Carriedo, A. (2020). Effects of an Interdisciplinary Approach Integrating Mathematics and Physical Education on Mathematical Learning and Physical Activity Levels. *JOURNAL OF TEACHING IN PHYSICAL EDUCATION*, *39*(1), 121–125. https://doi.org/10.1123/jtpe.2018-0274

Chaddock-Heyman, L., Erickson, K. I., Voss, M. W., Knecht, A. M., Pontifex, M. B., Castelli, D. M., Hillman, C. H., & Kramer, A. F. (2013). The effects of physical activity on functional MRI activation associated with cognitive control in children: A randomized controlled intervention. *Frontiers in Human Neuroscience*, *7*, 72–72. https://doi.org/10.3389/fnhum.2013.00072

Chaddock-Heyman, L., Weng, T. B., Kienzler, C., Weisshappel, R., Drollette, E. S., Raine, L. B., Westfall, D. R., Kao, S.-C., Baniqued, P., Castelli, D. M., Hillman, C. H., & Kramer, A. F. (2020). Brain network modularity predicts improvements in cognitive and scholastic performance in children involved in a physical activity intervention. *Frontiers in Human Neuroscience*, *14*. https://doi.org/10.3389/fnhum.2020.00346

Chaya, M. S., Nagendra, H., Selvam, S., Kurpad, A., & Srinivasan, K. (2012). Effect of yoga on cognitive abilities in schoolchildren from a socioeconomically disadvantaged background: A randomized controlled study. *J Altern Complement Med*, *18*(12), 1161–1167. https://doi.org/10.1089/acm.2011.0579

Cichy, I., Kaczmarczyk, M., Wawrzyniak, S., Kruszwicka, A., Przybyla, T., Klichowski, M., & Rokita, A. (2020). Participating in Physical Classes Using Eduball Stimulates Acquisition of Mathematical Knowledge and Skills by Primary School Students. *FRONTIERS IN PSYCHOLOGY*, *11*. https://doi.org/10.3389/fpsyg.2020.02194

Coe, D., Pivarnik JM, Womack CJ, Reeves MJ, & Malina RM. (2006). Effect of physical education and activity levels on academic achievement in children. *Med Sci Sports Exerc*, *38*(8), 1515–1519. https://doi.org/10.1249/01.mss.0000227537.13175.1b

Condello, G., Mazzoli, E., Masci, I., De Fano, A., Ben-Soussan, T. D., Marchetti, R., & Pesce, C. (2021). Fostering Holistic Development with a Designed Multisport Intervention in Physical Education: A Class-Randomized Cross-Over Trial. *International Journal of Environmental Research and Public Health*, *18*(18), 9871. https://doi.org/10.3390/ijerph18189871

Crova, C., Struzzolino I, Marchetti R, Masci I, Vannozzi G, Forte R, & Pesce C. (2014). Cognitively challenging physical activity benefits executive function in overweight children. *J Sports Sci*, *32*(3), 201–211. https://doi.org/10.1080/02640414.2013.828849

Dalziell, A., Booth, J. N., Boyle, J., & Mutrie, N. (2019). Better Movers and Thinkers: An evaluation of how a novel approach to teaching physical education can impact children's physical activity, coordination and cognition. *BRITISH EDUCATIONAL RESEARCH JOURNAL*, *45*(3), 576–591. https://doi.org/10.1002/berj.3514

Dalziell, A., Boyle, J., & Mutrie, N. (2015). Better Movers and Thinkers (BMT): An Exploratory Study of an Innovative Approach to Physical Education. *EJOP*, *11*(4), 722–741. https://doi.org/10.5964/ejop.v11i4.950

DeBruijn, A., Kostons DDNM, Van Der Fels IMJ, Visscher C, Oosterlaan J, Hartman E, & Bosker RJ. (2020). Effects of aerobic and cognitively-engaging physical activity on academic skills: A cluster randomized controlled trial. *J Sports Sci*, *38*(15), 1806–1817. https://doi.org/10.1080/02640414.2020.1756680

DeGreeff, A., Hartman E, Mullender-Wijnsma MJ, Bosker RJ, Doolaard S, & Visscher C. (2016). Long-term effects of physically active academic lessons on physical fitness and executive functions in primary school children. *Health Educ Res*, *31*(2), 185–194. https://doi.org/10.1093/her/cyv102

Donnelly, A., Hillman CH, Greene JL, Hansen DM, Gibson CA, Sullivan DK, Poggio J, Mayo MS, Lambourne K, Szabo-Reed AN, Herrmann SD, Honas JJ, Scudder MR, Betts JL, Henley K, Hunt SL, & Washburn RA. (2017). Physical activity and academic achievement across the curriculum: Results from a 3-year cluster-randomized trial. *Prev Med*, *99*, 140–145. https://doi.org/10.1016/j.ypmed.2017.02.006

D'Souza, A. A., & Wiseheart, M. (2018). Cognitive effects of music and dance training in children. *Archives of Scientific Psychology*, *6*(1), 178–192. https://doi.org/10.1037/arc0000048

Egger, F., Benzing V, Conzelmann A, & Schmidt M. (2019). Boost your brain, while having a break! The effects of long-term cognitively engaging physical activity breaks on children's executive functions and academic achievement. *PLoS One*, *14*(3), e0212482. https://doi.org/10.1371/journal.pone.0212482

Fedewa, A., Fettrow E, Erwin H, Ahn S, & Farook M. (2018). Academic-Based and Aerobic-Only Movement Breaks: Are There Differential Effects on Physical Activity and Achievement? *Res Q Exerc Sport*, *89*(2), 153–163. https://doi.org/10.1080/02701367.2018.1431602

Fedewa, A. L., Ahn, S., Erwin, H., & Davis, M. C. (2015). A randomized controlled design investigating the effects of classroom-based physical activity on children's fluid intelligence and achievement. *School Psychology International*, *36*(2), 135–153. https://doi.org/10.1177/0143034314565424

Fernandes, G. N. A., & Lemos, S. M. A. (2022). Quality of life and self-perceived health of adolescents in Middle School. *CoDAS*, *34*(6), e20210046. https://doi.org/10.1590/2317-1782/20212021046

Fisher, A., Boyle JM, Paton JY, Tomporowski P, Watson C, McColl JH, & Reilly JJ. (2011). Effects of a physical education intervention on cognitive function in young children: Randomized controlled pilot study. *BMC Pediatr*, *11*, 97. https://doi.org/10.1186/1471-2431-11-97

Frischen, U., Schwarzer, G., & Degé, F. (2019). Comparing the effects of rhythm-based music training and pitch-based music training on executive functions in preschoolers. *Frontiers in Integrative Neuroscience*, *13*. https://doi.org/10.3389/fnint.2019.00041

Gall, S., Adams L, Joubert N, Ludyga S, Müller I, Nqweniso S, Pühse U, du Randt R, Seelig H, Smith D, Steinmann P, Utzinger J, Walter C, & Gerber M. (2018). Effect of a 20-week physical activity intervention on selective attention and academic performance in children living in disadvantaged neighborhoods: A cluster randomized control trial. *PLoS One*, *13*(11), e0206908. https://doi.org/10.1371/journal.pone.0206908

Gallotta, M., Emerenziani, G. P., Iazzoni, S., & Guidetti, L. (2015). Impacts of coordinative training on normal weight and overweight/obese children's attentional performance. *Frontiers in Human Neuroscience*. https://doi.org/10.3389/fnhum.2015.00577

García-Hermoso, A., Hormazábal-Aguayo I, Fernández-Vergara O, González-Calderón N, Russell-Guzmán J, Vicencio-Rojas F, Chacana-Cañas C, & Ramírez-Vélez R. (2020). A before-school physical activity intervention to improve cognitive parameters in children: The Active-Start study. *Scand J Med Sci Sports*, *30*(1), 108–116. https://doi.org/10.1111/sms.13537

Giordano, G., & Alesi, M. (2022). Does Physical Activity Improve Inhibition in Kindergarteners? A Pilot Study. *Perceptual and Motor Skills*, *129*(4), 1001–1013. https://doi.org/10.1177/00315125221109216

Have, M., Nielsen JH, Ernst MT, Gejl AK, Fredens K, Grøntved A, & Kristensen PL. (2018). Classroom-based physical activity improves children's math achievement—A randomized controlled trial. *PLoS One*, *13*(12), e0208787. https://doi.org/10.1371/journal.pone.0208787

Hillman, C. H., Pontifex, M. B., Castelli, D. M., Khan, N. A., Raine, L. B., Scudder, M. R., Drollette, E. S., Moore, R. D., Wu, C.-T., & Kamijo, K. (2014). Effects of the FITKids Randomized Controlled Trial on Executive Control and Brain Function. *PEDIATRICS*, *134*(4), e1063–e1071. https://doi.org/10.1542/peds.2013-3219

Hraste, M., De Giorgio A, Jelaska PM, Padulo J, & Granić I. (2018). When mathematics meets physical activity in the school-aged child: The effect of an integrated motor and cognitive approach to learning geometry. *PLoS One*, *13*(8), e0196024. https://doi.org/10.1371/journal.pone.0196024

Hsieh, S., Lin CC, Chang YK, Huang CJ, & Hung TM. (2017). Effects of Childhood Gymnastics Program on Spatial Working Memory. *Med Sci Sports Exerc*, *49*(12), 2537–2547. https://doi.org/10.1249/MSS.0000000000001399

Jia, N., Zhang, X., Wang, X., Dong, X., Zhou, Y., & Ding, M. (2021). The Effects of Diverse Exercise on Cognition and Mental Health of Children Aged 5-6 Years: A Controlled Trial. *Frontiers in Psychology*, *12*, 759351. https://doi.org/10.3389/fpsyg.2021.759351

Kamijo, K., Pontifex, M. B., O'Leary, K. C., Scudder, M. R., Wu, C.-T., Castelli, D. M., & Hillman, C. H. (2011). The effects of an afterschool physical activity program on working memory in preadolescent children. *Dev Sci*, *14*(5), 1046–1058. https://doi.org/10.1111/j.1467-7687.2011.01054.x

Koutsandreou, F., Wegner, M., Niemmann, C., & Budde, H. (2015). Effects of motor vs. Cardiovascular exercise training on children's working memory. *Medicine and Science in Sports and Exercise*. https://www.researchgate.net/publication/288629976_Effects_of_motor_vs_cardiovascular_exercise_training_on_children's_working_memory

Kvalo, S., Edvin, B., Dyrstad, S. M., & Bonnick, K. (2017). Does Increased Physical Activity in School Affect Children's' Executive function and Aerobic Fitness? *Scandinavian Journal of Medicine and Science in Sports*, *27*(12). https://www.researchgate.net/publication/313817249_Does_Increased_Physical_Activity_in_School_Affect_Children's'_Executive_function_and_Aerobic_Fitness

Lakes, K. D., Bryars, T., Sirisinahal, S., Salim, N., Arastoo, S., Emmerson, N., Kang, D., Shim, L., Wong, D., & Kang, C. J. (2013). The Healthy for Life Taekwondo pilot study: A preliminary evaluation of effects on executive function and BMI, feasibility, and acceptability. *Mental Health and Physical Activity*, *6*(3), 181–188. https://doi.org/10.1016/j.mhpa.2013.07.002

Lakes, K. D., & Hoyt, W. T. (2004). Promoting self-regulation through school-based martial arts training. *Journal of Applied Developmental Psychology*, *25*(3), 283–302. https://doi.org/10.1016/j.appdev.2004.04.002

Leandro, C. R., Monteiro, E., & Melo, F. (2018). Interdisciplinary working practices: Can creative dance improve math? *RESEARCH IN DANCE EDUCATION*, *19*(1), 74–90. https://doi.org/10.1080/14647893.2017.1354838

Lin, J., Zhang, R., Shen, J., & Zhou, A. (2022). Effects of school-based neuromuscular training on fundamental movement skills and physical fitness in children: A systematic review. *PeerJ*, *10*, e13726. https://doi.org/10.7717/peerj.13726

Lind, R., Geertsen SS, Ørntoft C, Madsen M, Larsen MN, Dvorak J, Ritz C, & Krustrup P. (2018). Improved cognitive performance in preadolescent Danish children after the school-based physical activity programme 'FIFA 11 for Health' for Europe—A cluster-randomised controlled trial. *Eur J Sport Sci*, *18*(1), 130–139. https://doi.org/10.1080/17461391.2017.1394369

Marson, F., Fano, A. D., Pellegrino, M., Pesce, C., Glicksohn, J., & Ben-Soussan, T. D. (2021). Age-Related Differential Effects of School-Based Sitting and Movement Meditation on Creativity and Spatial Cognition: A Pilot Study. *Children*, *8*(7), 583. https://doi.org/10.3390/children8070583

Mavilidi, M. F., Lubans, D. R., Eather, N., Morgan, P. J., & Riley, N. (2018). Preliminary Efficacy and Feasibility of "Thinking While Moving in English": A Program with Physical Activity Integrated into Primary School English Lessons. *CHILDREN-BASEL*, *5*(8). https://doi.org/10.3390/children5080109

Mavilidi, M. F., Lubans, D. R., Miller, A., Eather, N., Morgan, P. J., Lonsdale, C., Noetel, M., Karayanidis, F., Shaw, K., & Riley, N. (2020). Impact of the 'Thinking while Moving in English' intervention on primary school children's academic outcomes and physical activity: A cluster randomised controlled trial. *International Journal of Educational Research*, *102*, N.PAG-N.PAG.

Mavilidi, M. F., & Vazou, S. (2021). Classroom-based physical activity and math performance: Integrated physical activity or not? *Acta Paediatrica (Oslo, Norway : 1992)*, *110*(7), 2149–2156. https://doi.org/10.1111/apa.15860

Mazzoli, E., Salmon, J., Pesce, C., Teo, W.-P., Rinehart, N., May, T., & Barnett, L. M. (2021). Effects of classroom-based active breaks on cognition, sitting and on-task behaviour in children with intellectual disability: A pilot study. *Journal of Intellectual Disability Research : JIDR*, *65*(5), 464–488. https://doi.org/10.1111/jir.12826

McMahon, S. D., Rose, D. S., & Parks, M. (2003). Basic Reading Through Dance Program: The Impact on First-Grade Students' Basic Reading Skills. *Eval Rev*, *27*(1), 104–125. https://doi.org/10.1177/0193841X02239021

Mead, T., Scibora, L., Gardner, J., & Dunn, S. (2016). The Impact of Stability Balls, Activity Breaks, and a Sedentary Classroom on Standardized Math Scores. *The Physical Educator*, *73*(3), 433–449. https://doi.org/10.18666/TPE-2016-V73-I3-5303

Meijer, A., Königs, M., van der Fels, I. M. J., Visscher, C., Bosker, R. J., Hartman, E., & Oosterlaan, J. (2021). The Effects of Aerobic Versus Cognitively Demanding Exercise Interventions on Executive Functioning in

School-Aged Children: A Cluster-Randomized Controlled Trial. *Journal of Sport & Exercise Psychology*, *43*(1), 1–13. https://doi.org/10.1123/jsep.2020-0034

Meloni, C., & Fanari, R. (2019). Chess Training Effect on Meta-Cognitive Processes and Academic Performance. *International Association for Development of the Information Society*. https://ezp.lib.cam.ac.uk/login?url=https://search.ebscohost.com/login.aspx?direct=true&db=eric&AN=ED608753&site=ehost-live&scope=site

Moreau, D., Kirk IJ, & Waldie KE. (2017). High-intensity training enhances executive function in children in a randomized, placebo-controlled trial. *Elife*, *6*. https://doi.org/10.7554/eLife.25062

Morris, J. L., Archbold, V. S. J., Bond, S. J., & Daly-Smith, A. (2022). Effects of Maths on the Move on Children's Perspectives, Physical Activity, and Math Performance. *Translational Journal of the American College of Sports Medicine*, *7*(1). https://doi.org/10.1249/TJX.0000000000000191

Mullender-Wijnsma, M., Hartman E, de Greeff JW, Bosker RJ, Doolaard S, & Visscher C. (2015). Improving academic performance of school-age children by physical activity in the classroom: 1-year program evaluation. *J Sch Health*, *85*(6), 365–371. https://doi.org/10.1111/josh.12259

Mullender-Wijnsma, M., Hartman E, de Greeff JW, Doolaard S, Bosker RJ, & Visscher C. (2016). Physically Active Math and Language Lessons Improve Academic Achievement: A Cluster Randomized Controlled Trial. *Pediatrics*, *137*(3), e20152743. https://doi.org/10.1542/peds.2015-2743

Mullender-Wijnsma, M. J., Hartman, E., de Greeff, J. W., Doolaard, S., Bosker, R. J., & Visscher, C. (2019). Follow-Up Study Investigating the Effects of a Physically Active Academic Intervention. *Early Childhood Education Journal*, *47*(6), 699–707.

Neville, R. D., & Makopoulou, K. (2020). Effect of a six-week dance-based physical education intervention on primary school children's creativity: A pilot study. *European Physical Education Review*, 1356336X2093958. https://doi.org/10.1177/1356336X20939586

Nobre, G. C., Nobre, F. S. S., & Valentini, N. C. (2022). Effectiveness of a Mastery Climate Cognitive-Motor Skills School-based Intervention in children living in poverty: Motor and Academic performance, self-

perceptions, and BMI. *Physical Education and Sport Pedagogy*, 1–17. https://doi.org/10.1080/17408989.2022.2054972

Norris, E., Dunsmuir S, Duke-Williams O, Stamatakis E, & Shelton N. (2018). Physically Active Lessons Improve Lesson Activity and On-Task Behavior: A Cluster-Randomized Controlled Trial of the 'Virtual Traveller' Intervention. *Health Educ Behav*, *45*(6), 945–956. https://doi.org/10.1177/1090198118762106

Oppici, L., Rudd, J. R., Buszard, T., & Spittle, S. (2020). Efficacy of a 7-week dance (RCT) PE curriculum with different teaching pedagogies and levels of cognitive challenge to improve working memory capacity and motor competence in 8–10 years old children. *Psychology of Sport and Exercise*, *50*, 101675. https://doi.org/10.1016/j.psychsport.2020.101675

Pesce, C., Crova, C., Marchetti, R., Struzzolino, I., Masci, I., Vannozzi, G., & Forte, R. (2013). Searching for cognitively optimal challenge point in physical activity for children with typical and atypical motor development. *Mental Health and Physical Activity*, *6*(3), 172–180. https://doi.org/10.1016/j.mhpa.2013.07.001

Pesce, C., Lakes, K. D., Stodden, D. F., & Marchetti, R. (2021). Fostering Self-Control Development With a Designed Intervention in Physical Education: A Two-Year Class-Randomized Trial. *Child Development*, *92*(3), 937–958. https://doi.org/10.1111/cdev.13445

Pesce, C., Masci, I., Marchetti, R., Vazou, S., Sääkslahti, A., & Tomporowski, P. D. (2016). Deliberate play and preparation jointly benefit motor and cognitive development: Mediated and moderated effects. *Frontiers in Psychology*, *7*. https://doi.org/10.3389/fpsyg.2016.00349

Reed, J., Einstein G, Hahn E, Hooker SP, Gross VP, & Kravitz J. (2010). Examining the impact of integrating physical activity on fluid intelligence and academic performance in an elementary school setting: A preliminary investigation. *J Phys Act Health*, *7*(3), 343–351. https://doi.org/10.1123/jpah.7.3.343

Resaland, G., Aadland E, Moe VF, Aadland KN, Skrede T, Stavnsbo M, Suominen L, Steene-Johannessen J, Glosvik Ø, Andersen JR, Kvalheim OM, Engelsrud G, Andersen LB, Holme IM, Ommundsen Y, Kriemler S, van Mechelen W, McKay HA, Ekelund U, & Anderssen SA. (2016). Effects of physical activity on

schoolchildren's academic performance: The Active Smarter Kids (ASK) cluster-randomized controlled trial. *Prev Med*, *91*, 322–328. https://doi.org/10.1016/j.ypmed.2016.09.005

Richard, V., Lebeau, J.-C., Becker, F., Boiangin, N., & Tenenbaum, G. (2018). Developing cognitive and motor creativity in children through an exercise program using nonlinear pedagogy principles. *Creativity Research Journal*, *30*(4), 391–401.

Riley, N., Lubans, D. R., Holmes, K., & Morgan, P. J. (2016). Findings From the EASY Minds Cluster Randomized Controlled Trial: Evaluation of a Physical Activity Integration Program for Mathematics in Primary Schools. *Journal of Physical Activity & Health*, *13*(2), 198–206. https://doi.org/10.1123/jpah.2015-0046

Riley N, Lubans DR, Morgan PJ, & Young M. (2015). Outcomes and process evaluation of a programme integrating physical activity into the primary school mathematics curriculum: The EASY Minds pilot randomised controlled trial. *J Sci Med Sport*, *18*(6), 656–661. https://doi.org/10.1016/j.jsams.2014.09.005

Rudd, J., Buszard, T., Spittle, S., O'Callaghan, L., & Oppici, L. (2021). Comparing the efficacy (RCT) of learning a dance choreography and practicing creative dance on improving executive functions and motor competence in 6–7 years old children. *Psychology of Sport and Exercise*, *53*, 101846. https://doi.org/10.1016/j.psychsport.2020.101846

Sánchez-López, M., Cavero-Redondo I, Álvarez-Bueno C, Ruiz-Hermosa A, Pozuelo-Carrascosa DP, Díez-Fernández A, Gutierrez-Díaz Del Campo D, Pardo-Guijarro MJ, & Martínez-Vizcaíno V. (2019). Impact of a multicomponent physical activity intervention on cognitive performance: The MOVI-KIDS study. *Scand J Med Sci Sports*, *29*(5), 766–775. https://doi.org/10.1111/sms.13383

Santos, S., Jiménez S, Sampaio J, & Leite N. (2017). Effects of the Skills4Genius sports-based training program in creative behavior. *PLoS One*, *12*(2), e0172520. https://doi.org/10.1371/journal.pone.0172520

Schmidt, S., Jäger K, Egger F, Roebers CM, & Conzelmann A. (2015). Cognitively Engaging Chronic Physical Activity, But Not Aerobic Exercise, Affects Executive Functions in Primary School Children: A Group-Randomized Controlled Trial. *J Sport Exerc Psychol*, *37*(6), 575–591. https://doi.org/10.1123/jsep.2015-0069

Sjöwall, D., Hertz, M., & Klingberg, T. (2017). No long-term effect of physical activity intervention on working memory or arithmetic in preadolescents. *Frontiers in Psychology*, *8*. https://doi.org/10.3389/fpsyg.2017.01342

Tanir, A., & Erkut, O. (2018). Effect of Rhythmic Basketball Lessons on Visual Attention Ability and Lay-Up Skill in School Children Aged 9-10. *Universal Journal of Educational Research*, *6*(9), 1857–1862.

Telford, R. D., Cunningham, R. B., Fitzgerald, R., Olive, L. S., Prosser, L., Xiaoli Jiang, & Telford, R. M. (2012). Physical Education, Obesity, and Academic Achievement: A 2-Year Longitudinal Investigation of Australian Elementary School Children. *American Journal of Public Health (1971)*, *102*(2), 368–374. https://doi.org/10.2105/AJPH.2011.300220

Telles, S., Singh, N., Bhardwaj, A. K., Kumar, A., & Balkrishna, A. (2013). Effect of yoga or physical exercise on physical, cognitive and emotional measures in children: A randomized controlled trial. *Child and Adolescent Psychiatry and Mental Health*, *7*. https://doi.org/10.1186/1753-2000-7-37

Tocci, N., Scibinetti, P., Mazzoli, E., Mavilidi, M. F., Masci, I., Schmidt, M., & Pesce, C. (2022). Giving Ideas Some Legs or Legs Some Ideas? Children's Motor Creativity Is Enhanced by Physical Activity Enrichment: Direct and Mediated Paths. *Frontiers in Psychology*, *13*, 806065. https://doi.org/10.3389/fpsyg.2022.806065

Tottori, N., Morita N, Ueta K, & Fujita S. (2019). Effects of High Intensity Interval Training on Executive Function in Children Aged 8-12 Years. *Int J Environ Res Public Health*, *16*(21). https://doi.org/10.3390/ijerph16214127

Tseng, W.-Y., Rekik, G., Chen, C.-H., Clemente, F. M., Bezerra, P., Crowley-McHattan, Z. J., & Chen, Y.-S. (2021). Effects of 8-Week FIFA 11+ for Kids Intervention on Physical Fitness and Attention in Elementary School Children. *Journal of Physical Activity & Health*, *18*(6), 686–693. https://doi.org/10.1123/jpah.2020-0823

Van de Niet, A., Smith J, Oosterlaan J, Scherder EJ, Hartman E, & Visscher C. (2016). Effects of a Cognitively Demanding Aerobic Intervention During Recess on Children's Physical Fitness and Executive Functioning. *Pediatr Exerc Sci*, *28*(1), 64–70. https://doi.org/10.1123/pes.2015-0084

Van den Berg, V., Saliasi, E., de Groot, R. H. M., Chinapaw, M. J. M., & Singh, A. S. (2019). Improving cognitive performance of 9–12 years old children: Just dance? A randomized controlled trial. *Frontiers in Psychology*, *10*. https://doi.org/10.3389/fpsyg.2019.00174

Van den Berg, V., Singh AS, Komen A, Hazelebach C, van Hilvoorde I, & Chinapaw MJM. (2019). Integrating Juggling with Math Lessons: A Randomized Controlled Trial Assessing Effects of Physically Active Learning on Maths Performance and Enjoyment in Primary School Children. *Int J Environ Res Public Health*, *16*(14). https://doi.org/10.3390/ijerph16142452

Van Klaveren, C., & De Witte, K. (2015). Football to Improve Math and Reading Performance. *Education Economics*, *23*(5), 577–595.

Vazou, S., & Skrade, M. A. (2017). Intervention integrating physical activity with math: Math performance, perceived competence, and need satisfaction. *International Journal of Sport and Exercise Psychology*, *15*(5), 508–522. https://doi.org/10.1080/1612197X.2016.1164226

Vetter, O'Connor HT, O'Dwyer N, Chau J, & Orr R. (2020). 'Maths on the move': Effectiveness of physically-active lessons for learning maths and increasing physical activity in primary school students. *J Sci Med Sport*, *23*(8), 735–739. https://doi.org/10.1016/j.jsams.2019.12.019

Watson, Timperio A, Brown H, & Hesketh KD. (2019). A pilot primary school active break program (ACTI-BREAK): Effects on academic and physical activity outcomes for students in Years 3 and 4. *J Sci Med Sport*, *22*(4), 438–443. https://doi.org/10.1016/j.jsams.2018.09.232

Zinelabidine, K., Elghoul, Y., Jouira, G., & Sahli, S. (2022). The Effect of an 8-Week Aerobic Dance Program on Executive Function in Children. *Perceptual and Motor Skills*, *129*(1), 153–175. https://doi.org/10.1177/00315125211058001
